# A 12-week in-phase bilateral upper limb exercise protocol promoted neuroplastic and clinical changes in people with relapsing remitting multiple sclerosis: A registered report randomized single-case concurrent multiple baseline study

**DOI:** 10.1371/journal.pone.0299611

**Published:** 2024-10-17

**Authors:** Dimitris Sokratous, Charalambos Costa Charalambous, Eleni Zamba—Papanicolaou, Kyriaki Michailidou, Nikos Konstantinou

**Affiliations:** 1 Department of Rehabilitation Sciences, Faculty of Health Sciences, Cyprus University of Technology, Limassol, Cyprus; 2 Physiotherapy Unit, Neurology Clinics, The Cyprus Institute of Neurology and Genetics, Nicosia, Cyprus; 3 Department of Neurology, Duke University School of Medicine, Durham, NC, United States of America; 4 Neuroepidemiology Department, The Cyprus Institute of Neurology and Genetics, Nicosia, Cyprus; 5 Biostatistics Unit, The Cyprus Institute of Neurology and Genetics, Nicosia, Cyprus; University of Rijeka Faculty of Medicine: Sveuciliste u Rijeci Medicinski fakultet, CROATIA

## Abstract

**Introduction:**

Relapsing-Remitting Multiple Sclerosis manifests various motor symptoms including impairments in corticospinal tract integrity, whose symptoms can be assessed using transcranial magnetic stimulation. Several factors, such as exercise and interlimb coordination, can influence the plastic changes in corticospinal tract. Previous work in healthy and chronic stroke survivors showed that the greatest improvement in corticospinal plasticity occurred during in-phase bilateral exercises of the upper limbs. Altered corticospinal plasticity due to bilateral lesions in the central nervous system is common after Multiple Sclerosis, yet the effect of in-phase bilateral exercise on the bilateral corticospinal plasticity in this cohort remains unclear. Our aim was to investigate the effects of in-phase bilateral exercises on central motor conduction time, motor evoked potential amplitude and latency, motor threshold and clinical measures in people with Relapsing-Remitting Multiple Sclerosis.

**Methods:**

Five people were randomized and recruited in this single case concurrent multiple baseline design study. The intervention protocol lasted for 12 consecutive weeks (30–60 minutes /session x 3 sessions / week) and included in-phase bilateral upper limb movements, adapted to different sports activities and to functional motor training. To define the functional relation between the intervention and the results, we conducted a visual analysis. If a potential sizeable effect was observed, we subsequently performed a statistical analysis.

**Results:**

Results demonstrated bilateral reduction of the motor threshold alongside with improvement of all clinical measures, but not in any other corticospinal plasticity measures.

**Conclusion:**

Our preliminary findings suggest that in-phase bilateral exercise affects motor threshold in people with Relapsing-Remitting Multiple Sclerosis. Therefore, this measure could potentially serve as a proxy for detecting corticospinal plasticity in this cohort. However, future studies with larger sample sizes should validate and potentially establish the effect of in-phase bilateral exercise on the corticospinal plasticity and clinical measures in this cohort.

**Trial registration:**

**Clinical trial registration**: ClinicalTrials.gov NCT05367947.

## Introduction

Multiple sclerosis (MS) is the most common inflammatory demyelinating and neurodegenerative disease of the central nervous system [1]. The global prevalence of MS during the last decade has increased by 30%, while the number of people suffering with MS worldwide is estimated at approximately 2.8 million [2]. The low average age of diagnosis (i.e., 32 years old), along with an average of seven years’ shorter life expectancy (i.e., 74.7 years) compared to the general population [3–5], highlights the need for a lengthy support, resulting in increased financial burden [6]. Recent studies reported that the annual mean cost of health care systems for people with MS living in Europe is about €40,000 [2]. Additionally, both MS patients and their caregivers, who usually are family members, face several psychological and social difficulties due to social isolation, poor quality of life, reduced productivity and lower general health levels [7, 8].

Relapsing-remitting MS (RRMS) is the most common type of MS and is characterised by periods of relapses followed by partial or complete recovery [9]. Inflammatory lesions are commonly found bilaterally in both white and grey matter of the central nervous system [10, 11], resulting in diverse clinical condition and symptoms, that include motor and cognitive impairments, visual deficits, depression and fatigue [11–13]. Those symptoms result in significantly low quality of life [14, 15], which subsequently cause the need for lifelong support and management of symptoms for most people with RRMS [16].

Motor symptoms in RRMS are associated with changes in corticospinal tract integrity and neuroplasticity [17–23]. The corticospinal tract is one of the major motor descending pathways providing voluntary motor function in humans [24]. The neuroplasticity of the corticospinal tract, is defined by changes in neuron structure or function, detected either directly from measures of individual neurons or inferred from measures taken across populations of neurons [25] and is an essential factor that predicts clinical recovery in the post-relapse stage of people with RRMS [26, 27]. Corticospinal plasticity can be probed using Transcranial Magnetic Stimulation (TMS) [28–30] and characterized via corticospinal excitability measures including resting motor threshold, motor-evoked potentials (MEPs) amplitude and latency, and the central motor conduction time (CMCT) [29]. Motor threshold and MEPs amplitude are the hallmark measures of corticospinal excitability in MS [31], whereas the MEPs latency and CMCT are temporal measures of the corticospinal excitability [32].

Corticospinal plasticity is exercise-dependent [33, 34] and influenced by various factors [35, 36], such as aerobic exercise [19, 37–39], resistance training [19, 39], as well as interlimb coordination [40, 41]. Previous studies that assessed corticospinal plasticity using TMS in healthy participants and in chronic stroke survivors, reported that interlimb coordination and especially in-phase bilateral movement has the strongest effect on corticospinal plasticity [42–45]. These effects are thought to be due to the suppression of cortical inhibition [43, 46] and the simultaneous activation of homologous representations of the motor cortices, which involves interhemispheric facilitation via transcallosal connection between the primary motor cortex and the supplementary motor area [47, 48].

Despite the broad literature on the effects of different types of exercises on the neuroplasticity in people with RRMS [38, 49–51], it is unclear whether in-phase bilateral exercises can promote motor related neuroplastic changes in RRMS. In light of evidence that people with RRMS have bilateral cortical lesions [52] which cause bilateral changes of corticospinal tract integrity [21, 23], these findings raise the question about the effects of in-phase bilateral exercises on corticospinal plasticity. Such effects would provide strong evidence about whether exercise, in particular in-phase bilateral exercise, can influence the corticospinal plasticity in RRMS.

The aim of this study was to investigate whether a 12-week intervention protocol of in-phase bilateral exercises for the upper limbs, which were adapted to sports activities and to functional training, could significantly affect the corticospinal plasticity and subsequently the individual clinical condition of people with RRMS. Our primary hypothesis was that a significant improvement of corticospinal plasticity would detect bilaterally, mainly in CMCT, caused by the specific intervention protocol which included in-phase bilateral exercises of the upper limbs, in people with RRMS. We assessed the corticospinal plasticity bilaterally using TMS and calculated corticospinal excitability measures [53]. Visual analysis was conducted separately for each variable and results are presented graphically according to the level, trend and stability, to define functional relationships between the intervention protocol and the corticospinal plasticity. Subsequently, a statistical analysis was performed in all outcome measures, which indicated a sizeable effect from the visual analysis, to estimate the effect of the intervention and then randomization tests were constructed to evaluate statistical significance [54].

Exploratory analyses in Stage 2 investigated the effects of the specific exercises protocol on resting motor threshold, the MEPs amplitude and latency, and on clinical symptoms using clinical assessments (i.e., gait, balance, strength, hand dexterity, cognitive functions, Modified Fatigue Impact Scale) [55].

The study followed a single-case concurrent multiple baseline design across subjects [56–58], which involved five people with RRMS. The specific design has the advantage to verify the cause-effect inference clearly by the staggered duration through separate baseline phases [59]. Consequently, we assumed that possible effects from our study would provide preliminary evidence and proof-of-concept evidence for this type of exercise, which can be applied during the disease progression and to existing neurorehabilitation protocols, in this particular clinical cohort.

## Materials and methods

### Participants

**Fig 1:**
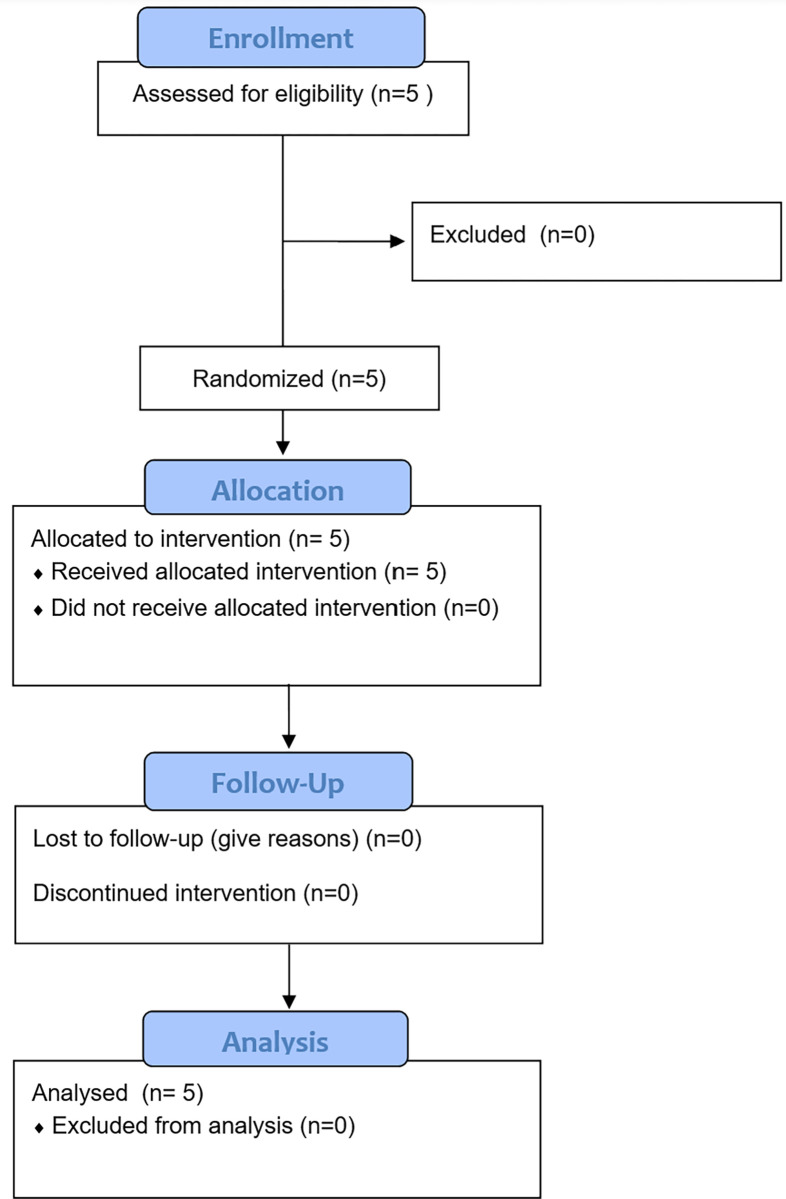
The CONSORT diagram. According to our study design (i.e., single case concurrent multiple baseline design across subjects), five participants were recruited and enrolled in the study based on the inclusion criteria. (n) number of participants. All of them finished the intervention protocol and all of them were included in data analysis.

All participants were recruited and evaluated by a neurologist at The Cyprus Institute of Neurology and Genetics from January to February 2023, and then they were randomly enrolled (Fig 1) by the neurologist and participated in the study from 10^th^ of March to 13^th^ of October 2023. Individual medical records were collected on 1^st^ of March 2023 and authors had not access to them so they couldn’t identify individual participants during or after data collection. The inclusion criteria included 1) diagnosis of RRMS, 2) Expanded Disability Status Scale score between three and five [60], 3) aged between 30 and 70 years, 4) no relapse within 30 days and 5) Mini Mental State of Examination score between 24 and 30 (no cognitive impairment) [61]. The exclusion criteria included 1) brain metal implants (e.g., titanium skull plates, aneurysm clips) [62], 2) history of any disease affecting the central nervous system other than MS (e.g., stroke, Parkinson`s disease, cerebral palsy), 3) history of cardiovascular disease (e.g., known aneurism, myocardial infarction, hyper/hypotension, heart failure), 4) mental disorders (e.g., depression, schizophrenia, bipolar syndrome), 5) severe orthopaedic disorders (e.g., knee or hip replacement, spondylosurgery, disk herniation, recent bone fracture), 6) pregnancy during the implementation of the study timeline, 7) visual deficit (e.g., optic neuritis, blindness, diplopia, glaucoma, blurred vision), 8) hearing impairments (i.e., deafness), 9) history of epileptic seizures and 10) spasticity level on upper or lower limbs more than 1+ (slight increase in muscle tone) according to Modified Ashworth Scale [63]. Additionally, participants were advised to continue their usual prescribed medication throughout the study duration, and they were advised to continue their usual routine and avoid receiving any other exercise program during the study. Furthermore, all participants read and signed a written informed consent, while all procedures were approved and conducted in accordance with the ethical guidelines of the Cyprus National Bioethics Committee before recruitment.

### Study design

**Fig 2:**
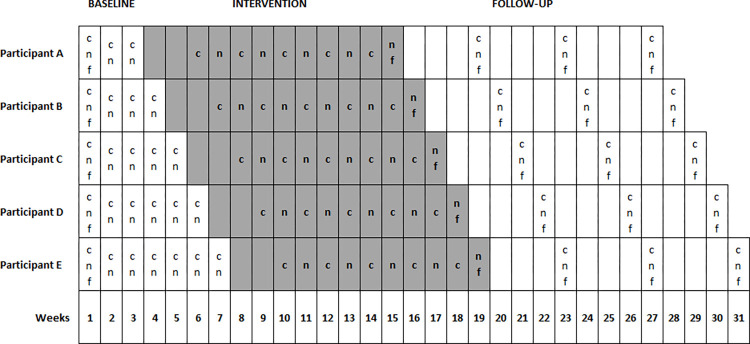
Timeline and schematic representation of the study’s design. Grey colour represents the intervention phase. Each row (A-E) represents a different participant. (c) clinical assessment. (f) Modified Fatigue Impact Scale questionnaire. (n) neurophysiological assessment via TMS. Every cell represents a different week, so every procedure which is included (i.e., c, n, f) was performed during the corresponding week but in different days.

The specific study is registered on ClinicalTrials.gov, with registration number NCT05367947. The study followed a single-case concurrent multiple baseline design across five subjects, without blinding and had been designed according to the “single case design” criteria, in which three participants [64], each with at least three data points per variable of interest across different phases is the minimum number needed to meet the standard criteria [58]. Therefore, we aimed to include five participants to ensure the reliability of the results in case of dropouts, as well as to record several data points across the baseline phase, five data points during the intervention phase and three data points in the follow up phase. During the experimental procedure, all participants began the study with the baseline phase at the same time while the intervention phase was introduced staggered across patients and time (Fig 2). The intervention was introduced systematically in one patient while baseline data collection continued in the others without any intervention. The cause-effect inference can be clearly verified by the staggered duration through separate baseline phases [59]. Subsequently, the intervention (i.e., in-phase bilateral exercises of the upper limbs) was the sole cause of improvement in participants’ conditions, the outcome measures did not change for the participants that remain in the baseline phase, but it was improved only for those in the intervention phase.

#### Baseline

As depicted in Fig 2, all patients began the baseline phase simultaneously. Each patient underwent a baseline phase of a different time duration (3–7 weeks), starting with three weeks for the first participant and gradually increased by one week for each participant. During the baseline phase, each participant was assessed on the Modified Fatigue Impact Scale [55] during the first week. The neurophysiological (i.e., CMCT, resting motor threshold, MEPs amplitude and latency) and clinical (i.e., gait, balance, strength, hand dexterity, cognitive functions) assessments were repeated after each baseline week for all participants.

#### Intervention

Immediately after the end of each baseline phase, the intervention phase began staggered across participants and time accordingly (Fig 2). The intervention protocol consisted of exercises based on in-phase bilateral movements of the upper limbs, which were adapted to different sport activities and to fitness functional exercises, organized in a circuit training considering the MS exercise recommendations [65]. Since no established protocols have been previously reported, for the needs of our study a certified fitness instructor designed these protocols adapted to different sport activities. Specifically, each session consisted of one to three sets, consisting of 20–30 repetitions of 9 different exercises targeting large muscle groups of the upper limbs (shoulder flexors, extensors, rotators, abductors and adductors, elbow flexors and extensors, hand and finger flexors and extensors). Additionally, three exercises targeted large lower limbs muscle groups (hip flexors, extensors, abductors and adductors, knee and ankle flexors and extensors) which were performed in between the upper limbs’ exercises and allowed relaxation of the upper limbs’ muscles.

The specific exercises included sports activities of basic technical skills of basketball (e.g., different types of passing, catching and throwing the ball) and volleyball (e.g., different types of passing and receiving the ball), whereas the fitness exercises included the diagonal movements from proprioceptive neuromuscular facilitation technique [66], as well as fingers flexion and extension by the use of a resistance hand training net [50]. To maintain the interest of the participants, the exercise program was modified throughout the course of the 12-week intervention period via changing the level of difficulty. For example, we used elastic bands with different resistance levels and different distance of the passes during the use of the ball.

The intervention phase for each participant consisted of 12 consecutive weeks in which the protocol was performed three times per week, for 30–60 minutes each session, adapted to each participant’s fatigue and fitness level. Each participant had to complete at least 27 (75%) out of 36 sessions for participant`s data to be included in the analysis [50]. Every intervention session consisted of a five minute warm-up (i.e., whole body range of motion exercises), followed by the main sport activities and fitness exercise protocol as described above, and a cool down for five minutes (i.e., passive stretching exercises of the muscle groups which are involved in the main part).

Additionally, starting from the third intervention week, we performed five neurophysiological and five clinical assessments (i.e., once a week), to collect five data points for every participant across the intervention phase. Moreover, each participant was asked to complete the Modified Fatigue Impact Scale (see secondary measures), once, at the end of the intervention phase [50] (Fig 2).

#### Follow-up

As depicted in Fig 2, every participant underwent three follow-up assessments in total, after finishing the training protocol, so to explore possible long-lasting effects. Each follow-up assessment included both neurophysiological and clinical measures. We performed the first follow-up assessment at the end of the fourth post-intervention week, the second one at the end of the eighth post-intervention week and the last follow-up assessment at the end of the 12^th^ post-intervention week (Fig 2).

### Primary outcome measure

Since prolongation of CMCT is the most common neurophysiological characteristic in people with MS [30, 67] and given the results of the study of Meng et al., [68], which indicated short term improvement of the CMCT after bilateral exercises of the upper limbs in stroke survivors, we designated the CMCT as our primary outcome variable. CMCT expresses the time taken for neural impulses to reach from motor cortex to alpha-motoneurons [67], which refer to the integrity of the white matter fibres [69]. Therefore, we calculated bilateral CMCT using both TMS and peripheral stimulation of the median nerve (*see below; Data Acquisition of Outcome Measures*) to observe possible changes in the central nervous system due to possible effects of the intervention protocol.

### Secondary outcome measures

The secondary outcome measures included the resting motor threshold (states the general excitability of the neuromotor axis in the target muscle), the MEPs amplitude (expresses the trans-synaptic activation of corticospinal neurons) and latency (defines the time which is needed for signal transmission from the motor cortex to the recording electrode of the target muscle) [70], and all clinical assessments. We quantified the resting motor threshold and the MEPs amplitude and latency using a single pulse TMS and two physiotherapists independently performed all clinical assessments to each participant.

### Data acquisition of outcome measures

We assessed the corticospinal plasticity using single pulse TMS in the neurophysiology lab of the Cyprus Institute of Neurology and Genetics. Using electromyography (EMG) signals from an upper limb muscle (see below; EMG recording), we collected MEPs, which were used to calculate all corticospinal excitability measures. During all neurophysiological assessments, participants were in a relaxed sitting position in a comfortable chair with feet touching the floor and both arms placed on cushioned armrests and with the head rested on a cushion. To ensure methodological consistency, we collected all data by performing the same methodological procedures for both conditions (i.e., corticospinal excitability measures bilaterally)—one side per assessment- across participants and across all time points.

#### EMG recording

During both TMS and peripheral stimulation, surface EMG of the Abductor Pollicis Brevis (APB) muscle was collected. We followed a standard skin preparation [71] and surface disk electrodes placement procedures by attaching the electrodes over the end plate region of the APB [72]. Specifically, the anode electrode was placed distally, whereas the cathode electrode proximally. A ground reference electrode was attached on the lateral condyle of the elbow, of the corresponding upper limb. Additionally, all signals were recorded with sampling rate of 24kHz and were filtered with a bandwidth of 2Hz–10 kHz using KeyPoint Net Software Electromyography (version 2.40; Natus Medical Incorporated G4, United States).

#### Peripheral stimulation

In addition to MEPs latency, calculating the CMCT requires two peripheral derived measures, the *F*- wave (i.e., late muscle response) and the *M*- wave (i.e., direct muscle response) [73, 74]. Therefore, we initially delivered peripheral stimulation on the median nerve at the wrist, approximately in an 8 cm distance from the cathode electrode [72], while collecting EMG from the APB [75].

#### TMS assessment

Following TMS recommended guidelines concerning safety and experimental conditions [70, 76], we assessed bilateral corticospinal excitability measures. We applied TMS single pulses [77] via figure-eight coil (C-B60; inner diameter: 35mm, outer diameter: 75mm), connected to the MagPro R20 (MagVenture User Guide, United Kingdom edition, MagVenture A/S, Denmark). The coil was oriented tangentially over the contralateral motor area of the brain, relative to the target muscle (i.e., APB), with a posterolateral handle pointing in approximately 45 degrees angle to the sagittal plane inducing posterior-anterior current in the brain [78].

For the TMS procedures, we first found the optimal stimulation site (i.e., hot-spot), next we determined the resting motor threshold and then we applied a bout of single pulses using suprathreshold stimulation. To determine hot-spot (i.e., the spot in which the largest response of the target muscle is elicited), we delivered single pulses at low intensities (e.g., ~20% maximum stimulator output) and gradually increased it by 1–5% maximum stimulator output until we reached the intensity that elicited three consecutive MEPs with peak-to-peak amplitude greater than 50mV [79, 80]. Then, we marked the position of the coil on the skull with a water-resistant ink, to determine the resting motor threshold of the target muscle. Resting motor threshold is the minimum stimulation intensity needed to produce MEPs of the target muscle. To identify the resting motor threshold, we employed an adaptive threshold-hunting method, the Motor Threshold Assessment Tool (MTAT 2.0) [81] (available at http://clinicalresearcher.org/software.htm). The specific method has the advantage of speed without losing accuracy when compared to the relative-frequency methods based on the Rossini–Rothwell, although both methods have similar precision [82, 83]. Then, to quantify the MEPs-derived measures of interest (i.e., MEPs amplitude and latency), we applied 30 suprathreshold stimuli [84] at 120% of the resting motor threshold [85].

#### Clinical assessment

We performed all clinical assessments in the physiotherapy unit of the Cyprus Institute of Neurology and Genetics. Two physiotherapists independently performed all clinical assessments to each participant, with the exact same methodological procedures, to ensure validity of the results [58]. However, the two assessors performed two clinical assessment trials to each participant prior to the beginning of the baseline phase, which were not included to the data analysis, but they were used as a training to the participants to eliminate variability of the outcome measures between the different baseline durations.

1. *Mini balance evaluation systems test*. It measures dynamic balance, functional mobility and gait in neurological patients, including people with RRMS [86]. The specific test consists of 14 items, including four of the six segments (anticipatory postural adjustments, sensory orientation, reactive postural control and dynamic gait) from the Balance Evaluation Systems Test. The Mini Balance Evaluation Systems Test is scored out of 28 points to include 14 items that are scored from zero to two.
2. *Six spot step test*. It is a timed walking test that involves kicking over a number of targets placed along a 5m-path in which rely to some extent on vision and cognition [87]. The Six Spot Step Test is measured in the time domain replicating a complex range of sensorimotor functions, part of which are lower limb strength, spasticity, coordination, as well as balance. We performed the specific test as described by Nieuwenhuis et al. [87] and recorded the mean time of the four runs as the final test result [88].
3. *Action research arm test*. It is a 19-item observational measure used by physiotherapists and other health care professionals to examine upper limb performance (i.e., coordination, dexterity and functioning) [89]. Items covering the Action Research Arm Test are categorized into four subscales (grasp, grip, pinch and gross movement) and arranged in order of decreasing difficulty, with the most difficult task examined first, followed by the least difficult task. The patient was sitting comfortable in front of a stable desk performing each task and the performance was rated on a four-point scale, ranging from 0 (no movement) to 3 (movement performed normally). We recorded the total score for each upper limb separately as the final test result.
4. *Isometric dynamometer*. We assessed the isometric muscle force of major muscle groups with the use of the muscle controller (Kinvent Biomechanique, Montpelier, France), which is a dynamometer used in the evaluation and rehabilitation of muscle strength that provides real time biofeedback [90]. The patient lied (supine or prone) on a therapeutic bed and the physiotherapist, with the use of the muscle controller, held against the patient’s limb as the patient exerted a maximal force. The physiotherapist countered the force (make test) or tried to break the contraction (break test) and the data were stored using the KFORCE APP (Kinvent Biomechanique, Montpelier, France). Shoulder flexors, extensors, rotators, horizontal adductors and abductors, elbow flexors and extensors are the major muscle groups which were evaluated. A separate value for each muscle group was recorded to be used in the data analysis.
5. *Symbol digit modalities test*. We employed the oral form of the test, which assesses the information processing speed [91]. During the test, the participant was given two minutes to orally match symbols with digits as quickly as possible. The key (specifying which symbols are assigned to which numbers) was located at the top of a computer screen. The researcher instructed the participants that each symbol is paired with a digit. Next, the participant was instructed to perform the test by responding orally to each symbol. For example, the symbol “O” is matched with the number “6”, so the correct response would be to say “six”. The researcher responsible for clinical assessments recorded the participant’s responses directly on a computer screen. The score was obtained by subtracting the number of errors from the number of items completed in two minutes.
6. *Modified fatigue impact scale*. It is a short questionnaire which requires the participants to describe the effects of fatigue during the past four weeks [55] (S1 Appendix). The Modified Fatigue Impact Scale consists of 21 questions which are subjectively rated from “0” (low rate) to “4” (high rate) and it is also divided into three subscales (i.e., physical, cognitive, and psychosocial). We recorded the total score of the test as the final test result. The higher the score is, the greater is the impact of fatigue in individual daily life. Therefore, we used the Modified Fatigue Impact Scale as the description of participants’ attribution of functional restrictions to fatigue symptoms.

## Analysis plan

To investigate possible effects of our protocol we followed recommended guidelines [92], in which we performed a separate analysis for each of the outcome measures, in all experimental phases (i.e., baseline, intervention and follow-up). We performed a visual analysis first, to determine whether there was a functional relationship between the intervention and the outcome measures. Then, a statistical analysis was performed in all outcome measures, which indicated a sizeable effect from the visual analysis, to evaluate the magnitude of the intervention effect [92].

### TMS measures analysis

Corticospinal plasticity was determined through changes of the corticospinal excitability measures. Hence, we quantified bilateral resting motor threshold, MEPs amplitude and latency, and CMCT, because each measure can assess different plastic changes across the neuromotor axis and they can be used as a proxy of corticospinal plasticity. Resting motor threshold (% maximum stimulator output) is the lowest intensity needed to elicit MEPs from a single-pulse TMS [76], amplitude (mV) is the difference in voltage between the maximal negative to maximal positive deflection of MEPs, which is referred as peak-to-peak amplitude [76], latency (ms) is the time between the TMS onset and the MEPs onset [67], while CMCT (ms) estimates the conduction time of corticospinal fibres between motor cortex and alpha-motoneurons [32].

For both upper limbs, corticospinal excitability measures (i.e., MEPs amplitude and latency) were first calculated from each MEP trace and then we calculated the mean to get a single value. These calculations were done according to the different time points for each participant in the baseline phase, at five time points in the intervention phase and at three time points in the follow-up phase (Fig 2). To investigate possible changes in corticospinal excitability, we measured resting motor threshold and calculated peak-to-peak amplitude throughout assessing MEPs [93] of the APB, while measuring of latency indicated possible changes in CMCT. Any changes in all measures across time points, indicated alterations in corticospinal plasticity [94]. We evaluated resting motor threshold using MTAT 2.0 [81] (available at http://clinicalresearcher.org/software.htm) and to investigate possible changes in individual corticospinal plasticity of each participant, we calculated bilaterally the difference between the mean values of each phase [78, 95]. On the other hand, from each stimulus response during the suprathreshold stimulation (i.e., 120% of resting motor threshold) [94], we calculated the MEPs peak-to-peak amplitude and latency, offline. To define CMCT (ms), we subtracted the peripheral conduction time ((*F-*wave latency + *M*-wave latency– 1)/2) from the MEPs latency. *F*-wave is the response of the targeted muscle produced by antidromic activation of motoneurons following the peripheral stimulation of motor nerve fibres, whereas *M*-wave produced by the direct muscle response [73–75]. A prolonged CMCT indicates damage of large fibres, demyelination of central motor pathways or slow summation of descending excitatory potentials in the corticospinal tract evoked by TMS [73, 96]. To standardise the latencies of all motor responses derived from different stimulation protocols (i.e., MEPs, *F-* and *M*- wave), we used a visual inspection from stimulation onset to response onset, performed from the same investigator so to ensure reliability and reproducibility of these measures across all time points. To define possible changes in CMCT, we evaluated the difference between the mean values of each phase bilaterally.

#### Clinical measures analysis

For each clinical measure (i.e., balance, gait, cognitive function, bilateral hand dexterity, strength and the Modified Fatigue Impact Scale) we calculated the mean values from each time point across all phases (i.e., baseline, intervention, follow-up), so to get a single mean value for each measure and for each phase (i.e., mean baseline, mean intervention, mean follow-up). To investigate the effect of the intervention protocol on the clinical condition, we calculated the differences between phases’ mean values (i.e., mean baseline, mean intervention, mean follow up), reflecting to the degree of the intervention-elicited change on the clinical condition following in-phase bilateral exercises.

### Visual analysis

Two independent assessors were systematically measured each outcome measure across time and inter-assessor agreement was calculated on at least twenty percent of the data points in each condition. The minimum acceptable inter-assessor agreement was set to 0.8 [58].

Initially, a visual analysis was conducted and data is presented graphically in spaghetti plots, to define whether there is a functional relation between the intervention and the outcome measures [92]. During the visual analysis, six features of the research design graphed data was examined: level, trend, stability, immediacy of the effect, overlap, and consistency. Over the within-phase examination an evaluation of level, trend and stability were examined. Level was reported from the mean score of each dependent variable and trend was determined as whether the data points are monotonically decreased or increased. To quantify the within phase differences in level and thus to identify whether there is substantial increase in the targeted behaviors, we used the Percentage of data Exceeding the Median (PEM) method [97]. Stability was estimated based on the percentage of data points falling within 15% of the phase median, if this was higher than 80% then we assume that this criterion was met. Additionally, over the between-phase examination an evaluation of overlapping data among baseline and intervention phases, consistency of data patterns and immediacy of effect were performed [92]. Immediacy of the effect was examined by comparing changes in level between the last three data points of one phase (e.g., baseline) and the first three data points of the next phase (e.g., intervention). Furthermore, consistency of data patterns involved the observation of the data from all phases within the same condition, with greater consistency expressing greater causal relation. Each feature was assessed individually and collectively across to all participants and all phases. Consequently, if the intervention protocol was the sole determinant of improvement, we expected to find indicators of improvement only at the intervention phase.

### Statistical analysis

A visual analysis was performed for each of the outcome variables to test for any effects due to the intervention. If the visual analysis indicated potential functional effects and met the six features (i.e., level, trend, stability, immediacy of the effect, overlap, and consistency) between baseline and intervention phase, we used the Nonoverlap of All Pairs (NAP) metric in order to estimate the effect of the intervention and randomization tests were constructed to evaluate statistical significance [54, 64, 96].

The null hypothesis was that there would be no improvement from the intervention protocol, thus participants’ responses are independent from the condition (baseline vs. intervention) under which they were observed. The alternative hypothesis was that the neurophysiological parameters and/or the clinical condition of the participants would be affected by the specific intervention, assessed separately. The null hypothesis was rejected if the *p*-value was smaller than the Bonferroni corrected *p*-value based on the actual number of tests that were performed (0.05/number of tests). All tests were two sided. Statistical analysis was performed using the statistical software R (https://www.r-project.org/).

### Possible threats

During the study implementation, different threats could be present and could affect internal validity of the study [58].

Attrition was one threat [58], which might had an impact on the experimental conditions in the case of less than three participants and less than three data points in each phase were presented [64]. Given that, we employed a specific methodology, which included five participants and at least three assessments points per participant, throughout all phases (i.e., baseline, intervention, follow-up) so to avoid attrition (Fig 2). Additionally, according to our protocol, participants had to complete at least 75% of the total intervention sessions, therefore this did not affect the implementation of our study in case of an absence during the intervention phase.

History is another possible threat [58]. Because we might had a limited ability to explore what other events would probably influenced the outcome measures, we asked from each participant to have a written calendar of their daily routine (e.g., any other physical activity, occupational and pharmaceutical changes) throughout the study duration. Also, by using the specific study design (i.e., single-case multiple baseline design) we eliminated the present of this thread, because we had the advantage to monitor and examine individual behaviour through the repetitive data collection during baseline and intervention phases. Moreover, to ensure that participants did not make other outcome-related changes in their daily life, they were advised prior to the study implementation to continue their usual prescribed medication throughout the study duration. However, none of the participants made any changes to their usually prescribed medication upon physician recommendation.

## Results

Following our sampling plan [98], a total of five participants were recruited following the inclusion/exclusion criteria. All participants completed all assessments (Fig 2) and the exercise protocol without complaints or side effects. Participant E missed one assessment data point at baseline and another assessment during the intervention phase. Despite these omissions, Participant E’ data were retained and included in the overall analysis. All data can be found in FIGSHARE repository (https://doi.org/10.6084/m9.figshare.26527351.v1). Demographics and baseline clinical characteristics of participants were collected prior to the intervention and are presented in Table 1.

**Table 1:** Participants’ demographic and baseline clinical characteristics.

Participant	A	B	C	D	E
Age (years)	56	56	47	52	59
Sex	Female	Female	Female	Female	Female
Dominant hand	Right	Right	Right	Right	Right
Weight (kg)	83	57	50	66	55
Height (m)	1.68	1.58	1.55	1.68	1.60
BMI	29.4 (overweight)	22.8 (normal)	20.8 (normal)	23.4 (normal)	21.5 (normal)
HR^a^ max (bpm)	164	164	173	168	161
Left upper limb length^b^ (cm)	69	61	60	68	67
Right upper limb length^b^ (cm)	69	61	60	68	67
EDSS	3	3.5	3.5	3	3.5
Disease duration (years)	9	7	3	9	8
Current clinical symptoms	• General weakness. • Minor imbalance during gait.	• General weakness. • Minor imbalance during gait. • Spasticity: grade one on both feet according to the Modified Ashworth Scale [63].	• General weakness. Minor imbalance during gait and standing. • Spasticity: grade one on both feet according to the Modified Ashworth Scale [63].	• General weakness. • Minor imbalance during gait.	• General weakness. • Minor imbalance during gait.
MMSE [61]	30 (no cognitive impairment)	30 (no cognitive impairment)	30 (no cognitive impairment)	30 (no cognitive impairment)	30 (no cognitive impairment)
MS related medication	Gilenya [99, 100]	Gilenya [99, 100]	Ocrelizumab [101]	Gilenya [99, 100]	Aubagio [102]
Occupation	Sedentary	Sedentary	Sedentary	Sedentary	Sedentary

BMI, Body Mass Index; HR, Heart Rate; EDSS, Expanded Disability Status Scale; MMSE, Mini Mental State Examination; MS, Multiple Sclerosis. All participants were right-handed, in the same decade of age and with similar body characteristics. Because upper limb length is a factor contributing to MEPs responses [103], both upper limbs`length were measured for all participants, without a difference between both sides. EDSS score for all participants was between 3 and 3.5, which indicating moderate disability [60]. The MMSE and current clinical symptoms were in line with inclusion/exclusion criteria, so there was no specific impact related to the TMS and clinical measures. All participants were engaged in sedentary work [104, 105] which required low physical demands and no frequent moves; thus it may not have an impact on the study`s outcome measures.

^a^Heart Rate maximum was calculated from the equation 220-age [106].

^b^Upper limbs length were measured in an anatomical position from C7 spinous process to the ulnar head [107].

The exercise protocol lasted 12 consecutive weeks and contained three sets of 12 exercises within each set, with two minutes rest between the sets. All details regarding the exercise protocol are presented in Table 2.

**Table 2:** Overview of the exercise protocol.

	Type of exercise	Repetitions	Body position	Difficulty level
1	Basketball chest pass	20–30	Standing	Distance of the pass
2	PNF 1st diagonal FP	20–30	Standing	Elastic band
3	Flexion of all fingers	20–30	Sitting	Hand training net
4	Adductors squeeze	20–30	Supine lying	Pilates ring
5	Basketball shoulder pass	20–30	Standing	Distance of the pass
6	PNF 1st diagonal EP	20–30	Standing	Elastic band
7	Extension of all fingers	20–30	Sitting	Hand training net
8	Hips Abduction	20–30	Supine lying	Pilates ring
9	Volleyball overhead pass	20–30	Standing	Distance of the pass
10	PNF 2nd diagonal FP	20–30	Standing	Elastic band
11	PNF 2nd diagonal EP	20–30	Standing	Elastic band
12	Squat	20–30	Standing	Balance pads

PNF, Proprioceptive Neuromuscular Facilitation; FP, Flexion Pattern; EP, Extension Pattern; Each session included three sets of nine different exercises which targeted large muscle groups of the upper limbs (i.e., 1–3, 5–7, 9–11) and three exercises which targeted large muscle groups of the lower limbs (i.e., 4, 8, 12). Overall, for all participants the range of repetitions was 20–30 according to individuals’ fitness level. The difficulty level for the sport activities (i.e., 1, 5, 9) was maintained by changing the distance of the passes. The difficulty level for the exercises of the hip adduction and abduction (i.e., 4 and 8) was maintained by changing the resistance of the Pilates ring, whereas for the squats (i.e., 12) the difficulty level was sustained by changing the base of support (e.g., balance pads, Bosu ball). The difficulty level for the strengthening of the fingers (i.e., 3 and 7) was maintained by changing the resistance of the hand training net. Finally, all PNF exercises (i.e., 2, 6, 10, 11) were performed against the resistance of elastic bands (different resistance accordingly), which it was attached by a stable point.

For the duration of the intervention implementation period, we had continuous monitoring and record of the participants’ performance. Individual performance data are presented in Table 3.

**Table 3:** Individual performance during the exercise protocol.

Participant	A	B	C	D	E
Sessions Completed	32/36	31/36	32/36	32/36	34/36
Number of Repetitions	821	824	945	956	773
HR (bpm)	93	108	102	94	101
%HR maximum	57	66	62	56	63
RPE	4	4	5	5	3
Body Temperature (°C)	36.1	36.3	35.3	35.7	36.2
Resistance Level	Medium	Light	Medium	Medium	Medium

HR, Heart Rate; RPE, Rating of Perceived Exertion; Individual mean values from the total completed number of sessions are presented in Table 3. All participants completed more than 75% (group mean; 89%, 32/36 sessions) of the total intervention sessions, which was set as the minimum accepted percentage of completed sessions per participant. Furthermore, all participants exceeded the recommended number (i.e., 300) of repetitions required in a session to induce neuroplastic effects [108]. Additionally, all of them completed the exercise protocol following the recommended exercise features regarding HR, RPE, body temperature and resistance level [65]. However, the percentage of the maximum HR indicated that none of them exceeded the aerobic level of exercise (i.e., below 70% of each participant`s maximum HR) [109] during the sessions. In summary, for all participants the range of the exercise HR was 56% - 66% of the individual maximum HR, the range of RPE [110] was 3–5 (on a 10-point scale) and the range of body temperature during exercise was 35.3°C - 36.3°C.

Since our main aim was to induce neuroplasticity as an effect of the specific type of movement (i.e., in-phase bilateral movement of the upper limbs) and not as a training effect [37, 39, 111, 112], we maintained individual performance under the Heart Rate (HR) zone of aerobic exercise (i.e., below 70% of each participant`s maximum HR [109]). To keep a constant individual HR and body temperature, at the end of each set across sessions, we used a pulse oximeter (ChoiceMMed OxyWatch C29, Bristol, United States) for the HR screening and a forehead thermometer to monitor the body temperature. The room temperature was controlled at 24°C, so all participants were always exercising at the same temperature.

### Primary outcome measure—central motor conduction time

A decrease in CMCT signifies an improvement. The results of the CMCT assessments during all time points are presented in Table 1 in S2 Appendix.

#### Within-phase visual analysis

Data for both left and right upper limbs were analyzed in terms of variability (i.e., the spread of data points within a phase that was indicated from the two-standard deviation band, which is the mean of a phase and adding and subtracting two standard deviations from it [64]), stability [113] and trend. Low variability (i.e., 0–20%), was observed for all participants except for Participant E who showed high variability on both the baseline (50%) and the intervention (40%) phase for the left upper limb, whereas high variability (40%) was observed for the right upper limb during the baseline phase, but not during the intervention phase (0%).

Stability of the data (>80% [92]), was observed for Participants A (baseline: 100%, intervention: 80%) and C (baseline: 80%, intervention: 80%) for the left upper limb. Participants B (intervention: 100%), C (baseline: 80%, intervention: 100%) and E (intervention: 80%) were the only ones who met the stability criterion for the right upper limb.

In terms of the trends of the CMCT, during the baseline phase all directions must be stable, whereas during the intervention phase downward or stable (given that there is a change in level between phases) directions signify an improvement. During the baseline phase, three out of five participants (B, D, E) showed downward trends, whereas two out of five (A, C) showed upward trends for the left upper limb. During the intervention phase, Participants A, B, C and D showed downward trends, while Participant E showed a stable trend for the left upper limb. On the other hand, during the baseline phase only Participant B showed stable trend, while Participants A and C showed downward trends and Participants D and E showed upward trends, for the right upper limb. However, during the intervention phase three out of five participants (A, C, D) showed downward trends, while Participants B and E showed downward and stable trends respectively for the right upper limb. As a conclusion, none of the participants met all the within-phase visual analysis’ criteria, for either upper limb.

#### Between-phases visual analysis

Data from both baseline and intervention phases were included in the between-phases analysis. The criteria which were used for the visual analysis, were level (i.e., change of mean values between phases), immediacy (i.e., change in level between the last three data points of the baseline phase and the first three data points of the intervention phase) and the PEM [97]. Reduction (i.e., improvement) of mean values (Table 1 in S2 Appendix) was observed for Participants C (baseline: 13 ms, intervention: 11 ms) and D (baseline: 14.2 ms, intervention: 11.6 ms), for the left upper limb, while participants A (baseline: 7.7 ms, intervention: 7.3 ms), B (baseline: 11.4 ms, intervention: 8.5 ms), C (baseline: 12.1 ms, intervention: 11.9 ms) and E (baseline: 9.3 ms, intervention: 7.1 ms), showed reduction of mean values for the right upper limb. Therefore, participants C and D showed a difference in level for the left upper limb, though Participants A, B, C and E showed a difference in level and immediate effect for the right upper limb. The PEM [97] indicates an effect when 70% of data of the intervention phase exceed the median of the baseline phase. According to our results, only Participant C (PEM = 80%) showed an effect for the left upper limb of, whereas Participants B (PEM = 100%) and E (PEM = 80%) showed an effect for the right upper limb. In summary, none of the participants met all visual analysis’ criteria [92]; therefore, we did not proceed with statistical analysis of those data, in line with our pre-registered analysis plan.

### Secondary outcome measures

#### TMS assessment

Throughout the within and between-phases visual analysis, no evidence of improvement related to MEPs amplitude and latency was observed for any of the participants. The results of the MEPs amplitude and latency assessments during all time points are presented in Tables 2 and 3 in S2 Appendix.

#### MEPs amplitude

An increase in MEPs amplitude signifies an improvement. Only Participant D (baseline mean = 0.08mV, intervention mean = 0.1mV) showed an improvement for the left upper limb, whereas Participants A (baseline mean = 0.47mV, intervention mean = 0.1mV) and B (baseline mean = 0.09mV, intervention mean = 0.1mV) showed an improvement for the right upper limb. Participants A (left upper limb), B (left upper limb) and D (right upper limb) showed an improvement of MEPs amplitude, but low data stability was observed on both baseline and intervention phases for these three participants (A: baseline = 33%, intervention = 40%; B: baseline = 0%, intervention = 40%; C: baseline = 33%, intervention = 40%).

#### MEPs latency

Reduction in MEPs latency signifies an improvement. Three out of five participants (C: baseline mean = 26.4 ms, intervention mean = 24.5 ms; D: baseline mean = 29.6 ms, intervention mean = 26.6 ms; E: baseline mean = 23.5 ms, intervention mean = 22.6 ms) showed an improvement on the left upper limb measures, whereas four out of five participants (A: baseline mean = 23.6ms, intervention mean = 21.7ms; B: baseline mean = 24.6ms, intervention mean = 21.7ms; C: baseline mean = 25.1ms, intervention mean = 24.9ms; E: baseline mean = 22.9ms, intervention mean = 21.6ms) showed an improvement on the right upper limb measures.

Even though an improvement was observed in the mean values, low data stability and unexpected trend directions were detected. To meet the stability criterion, the data on any of the phases needs to be more than 80% and to meet the trend criterion, the expected directions needs to be stable during the baseline and downward or stable stable (given that there is a change in level between phases) during the intervention phase [92]. Participant C showed an upward trend during baseline, Participant D showed a downward trend and low data stability (66%) during baseline and Participant E showed an upward trend and low data stability (60%) during the intervention, for the left upper limb. Participants A and C showed a downward trend during baseline, Participant B showed an upward trend during baseline and Participant E showed low data stability (66%) during baseline, for the right upper limb. Therefore, none of the participants could be included for statistical analysis for the MEPs amplitude and latency.

#### Resting motor threshold

**Fig 3:**
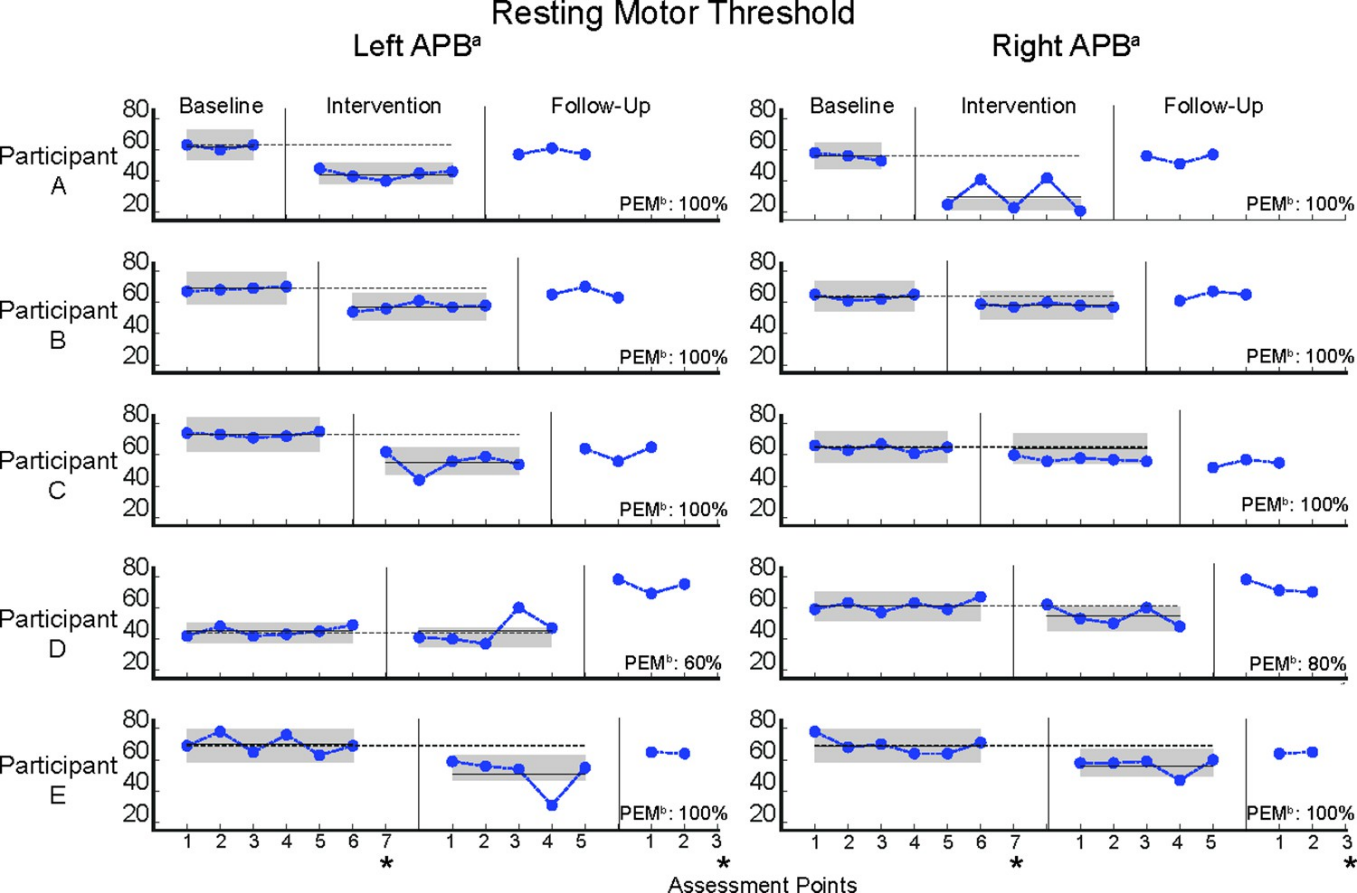
Visual representation of the resting motor threshold during baseline, intervention and follow-up phases. (APB) Abductor Pollicis Brevis. (PEM) Percentage Exceeding the Median. Data of each participant are presented regarding left and right upper limb, in terms of left and right APB. The number of assessment points per phase are presented on the x-axis, whereas on the y-axis the values of the resting motor threshold are presented. Resting motor threshold, is measured by means of the percentage of the Maximum Stimulator Output (%MSO). The vertical lines between the data points indicate the three study phases (i.e., baseline, intervention, follow-up). The grey area around the data points, refers to the acceptable range regarding the stability criterion (i.e., ±15% of the median of each phase [92]). The black horizontal dashed lines represent the PEM [97]. The black horizontal lines represent the within-phase mean. Although, the mean lines for all baseline phases may not be clearly visible as they are superimposed with the PEM lines. While on the intervention phase the mean lines are well seen, thus indicating the effect of the intervention (except that of Participant D; left APB). Also, some data points during the intervention phase may be detected to be outside of the data stability range (Participant C: second intervention point for the left APB and Participant E: fourth intervention point bilaterally), yet the percentage of the within-phase stability for them is greater than the accepted 80% [92]. (*) denotes the missed assessment points, which Participant E couldn’t perform (i.e., last TMS assessment of baseline and follow-up phases bilateral). During the follow-up phase all data showed a tendency to return towards the baseline level. ^a^Data from both left and right Abductor Pollicis Brevis (APB) muscle was collected. ^b^The Percentage Exceeding the Median has been calculated between the baseline and intervention phases only.

A decrease in resting motor threshold signifies an improvement. The repeated assessments of the resting motor threshold across all assessment time points are depicted in Fig 3.

*Within-phase visual analysis*. Data were analyzed in terms of variability [64], stability [113] and trend for both left and right upper limbs. All participants showed no variability during both baseline and intervention phases, for the left upper limb. The criterion of data stability and the expected trend direction (i.e., baseline: stable trend, intervention: downward or stable trend given that there is a decreased level compared to baseline phase, follow-up: upward or stable trend given that there is a decreased level compared to baseline phase) was met from Participants A (baseline: stability = 100%, stable trend; intervention: stability = 100%, stable trend), B (baseline: stability = 100%, stable trend; intervention: stability = 100%, stable trend), C (baseline: stability = 100%, stable trend; intervention: stability = 100%, stable trend) and E (baseline: stability = 100%, stable trend; intervention: stability = 80%, downward trend), for the left upper limb. Although, Participant D showed data stability on both baseline (100%) and intervention (80%) phases, upward trends (i.e., unexpected direction) presented on both baseline and intervention phases, for the left upper limb. Therefore, all participants met all criteria from the within-phase visual analysis for the left upper limb, except from Participant D who showed unexpected trends directions.

No variability for all participants was observed on both baseline and intervention phases for the right upper limb. However, only Participants B (baseline: stability = 100%, stable trend; intervention: stability = 80%, downward trend), C (baseline: stability = 100%, stable trend; intervention: stability = 100%, downward trend) and E (baseline: stability = 100%, stable trend; intervention: stability = 80%, downward trend) met the criteria of data stability and trend, for the right upper limb. Although Participant A showed data stability during the baseline phase (100%), low data stability (60%) was observed during the intervention phase for the right upper limb. Also, Participant D showed data stability on both baseline (100%) and intervention (80%) phases, but an upward trend (i.e., unexpected direction) was observed during the baseline phase, for the right upper limb. Therefore, all participants met all criteria from the within-phase visual analysis for the right upper limb, except from Participants A who showed low data stability during the intervention phase and from Participant D, who showed an unexpected trend direction (i.e., upward) during the baseline phase. Consequently, Participants A, B, C and E met all the within-phase criteria for the left upper limb, whereas Participants B, C and E met all criteria for the right upper limb.

*Between-phases visual analysis*. Resting motor threshold data from both the baseline and the intervention phase were included in the between-phases analysis. The criteria used for the data analysis included the proportion of data overlap, level, immediacy and the PEM [97].

No data overlapping was observed for any participant, except for Participant D, who showed 40% of data overlapping for the left upper limb and 60% overlapping for the right upper limb. As shown in Fig 3, Participants A, B, C and E showed reduction of mean values for both left and right upper limbs, while Participant D showed reduction of mean value only for the right upper limb.

All participants showed immediacy of the effect for both left and right upper limb, except for Participant D for the left upper limb. Moreover, the PEM data indicated that Participants A, B, C and E showed high effectiveness (100%) [97] for both left and right upper limb. Participant D showed a moderate effect (PEM = 80%) only for the right upper limb, while no effectiveness (PEM = 40%) was found for the left upper limb. Participant D was the only one who did not satisfy the between-phases criteria.

In summary, throughout the within and between phases visual analyses, all criteria were met by Participants A, B, C and E for the left upper limb and by Participants B, C and E for the right upper limb.

Following our registered analysis plan, only the data from the participants who met the criteria from the visual analysis were included in statistical analysis [58, 92]. As was indicated from the NAP index, Participant A (NAP = 1, *p* < 0.05) and Participants B, C and E (NAP = 1, *p* < 0.01) showed significant improvement of the resting motor threshold, for the left upper limb. Moreover, Participants B, C and E (NAP = 1, *p* < 0.01) showed significant improvement, for the right upper limb. The results of Participant A were not significant following a Bonferroni correction of 0.05/2, since we tested the same value for both upper limbs.

In general, four out of five participants (A, B, C, E) showed an improvement on the left upper limb measures, whereas three out of five participants (B, C, E) showed an improvement on the right upper limb measures. During the baseline phase, higher resting motor threshold for the left upper limb was observed (group mean = 64% Maximum Stimulator Output; MSO), compared to the right upper limb (group mean = 62% MSO). During the intervention phase we found lower resting motor threshold for the left upper limb (group mean = 50% MSO), compared to the right upper limb (group mean = 52% MSO).

For the follow-up phase, a descriptive analysis was performed for all participants, indicating an increase of the resting motor threshold for both left and right upper limbs. As was indicated from both left and right upper limbs mean values (left upper limb: group mean = 65% MSO; right upper limb: group mean = 62% MSO), all participants showed a trend to return in baseline values.

#### Clinical assessment

The analysis indicated an improvement on all clinical assessments (i.e., Mini Balance Evaluation System Test, Six Spot Step Test, Action Research Arm Test, Isometric Dynamometer test, Symbol Digit Modalities Test, Modified Fatigue Impact Scale) and a high level of agreement (percentage of agreement = 1) in three out of five clinical assessments (Mini Balance Evaluation System Test, Six Spot Step Test, Action Research Arm Test), across all study phases. The details and results of all phases are included in the Supplementary Material (Tables 1–4 in S3 Appendix). All criteria of the visual analysis [92] were satisfied for both within and between-phases analysis for all the participants, for both left and right upper limbs. Therefore, all participants and all clinical outcome measures were included in the statistical analysis.

*Within-phase visual analysis*. Data for both left and right upper limbs were analyzed in terms of variability [64], stability [113] and trend. All participants showed no variability [64], high data stability (100%) [113] and expected trends directions (baseline: stable trend; intervention: downward or stable trend given that there is a decreased level between phases, Follow-up: upward or stable trend given that there is a decreased level compared to baseline phase).

*Between-phases visual analysis*. Data form baseline and intervention phases were included in the between-phases analysis. The criteria used for the data analysis included level, proportion of data overlap, immediacy and the PEM [97]. All participants showed a change (i.e., improvement) in level, with no data overlapping between phases for both left and right upper limbs. Also, all participants showed an immediate effect and a high level of effectiveness (PEM = 100%) [97], for both left and right upper limbs. However, during the follow-up phase, all participants showed a minor reduction (i.e., decrease of the values) of the individual performance compared to the intervention phase, as was indicated throughout the individual data visual description.

Since all participants met all visual analysis criteria, they were all included in the statistical analysis, for all clinical outcome measure. The NAP index was used, which indicated significant results for all participants (NAP = 1, *p* < 0.05). The results for all clinical measures are presented in Tables 1–4 in S3 Appendix. Although the results indicated nominal statistical significance, these would not survive a Bonferroni correction, if corrected for the total number of clinical tests performed. Nevertheless, this is an important observation in our pilot study that warrants further investigation.

## Discussion

This investigation was designed to determine the influence of an in-phase bilateral upper limb exercise protocol on corticospinal plasticity, specifically its effect on CMCT, and to assess its secondary impact on clinical outcomes in individuals with RRMS. While our hypothesis anticipated that the exercise protocol would enhance corticospinal plasticity, as reflected decrease of CMCT, the findings were only partially congruent with this prediction.

Contrary to expectations, the exercise protocol did not result in significant changes in CMCT. However, the intervention did have a notable impact on the resting motor threshold—a key indicator of cortical excitability and plasticity [114, 115]. This outcome implies that while the exercises did not alter neural conduction speed, they may have facilitated increased neural excitability. In addition to these neurophysiological insights, the protocol led to observable improvements in clinical measures. These enhancements spanned various domains, including motor function and cognitive processing, suggesting a broad therapeutic potential of the exercise regimen for individuals with RRMS.

The partial support of our hypothesis underscores the complexity of modulating corticospinal plasticity and highlights the need to further investigate the mechanisms through which exercise affects neurophysiological parameters in this clinical population. It also points to the potential for targeted exercise interventions to yield clinically meaningful improvements, even in the absence of detectable changes in conduction velocity (i.e., CMCT).

### Exercise effect on CMCT, MEPs amplitude and latency

Following visual analysis of the CMCT measurements, no significant improvement (i.e., reduction) was observed to any of the participants, possibly due to high variability and low data stability across all study phases. Given that participants did not engage in any exercise during baseline, we expected to observe stability of the data during the baseline phase. Conversely, we expected an exercise-induced effect on CMCT during the intervention phase. During the follow-up phase, we expected no significant improvement because participants did not engage in an exercise regime during this period. Although our findings indicated an observable stable baseline for the right (Participants A, C, E) and left (Participants B, E) upper limbs, there was no improvement during the intervention on either the left or the right side.

The current study employed a single-case concurrent multiple baseline design with a small number of participants (*n* = 5), and the small sample size could be a reason for not detecting a CMCT effect in any of the participants. The participants (i.e., C and D for the left upper limb; A, B, C and E for the right upper limb) who showed an improvement (i.e., reduction of CMCT) between baseline and intervention phases, also showed unexpected trend directions (i.e., upward/downward) within study phases.

The observed variability in trend directions, likely stemming from inconsistent data across the study phases, may be attributed to factors such as manual coil positioning without neuronavigation system [116] and the use of varying TMS intensities (i.e., resting motor threshold was assessed on each session) per session [117]. Also, the variability of TMS intensities used in each session could be another reason for such low data stability. For example, Pellegrini et al. [116] discuss that different TMS intensities may activate different corticospinal pathways, whereas Di Lazzaro and Rothwell [118] indicated that high stimulus intensities result to later descending volleys (i.e., I waves). Another contributor to low data stability could be the fact that corticospinal integrity in some people with MS can be affected during the very early disease stage [119], resulting in slower or blockage of the conduction time (i.e., prolonged CMCT) [120].

Furthermore, no effect was found in MEPs amplitude and latency, possibly due to low data stability (i.e., < 80%) and due to unexpected trend directions (i.e., upward/downward). It is well established that MEPs amplitude is highly variable between-subjects and within-subjects in the same trial or on repeated trials [121–123]. Another possible physiological explanation for not observing differences in the values of MEPs amplitudes and latencies is the high resting motor threshold. For example, Caramia et al. [124] observed that individuals with a high resting motor threshold exhibited altered MEPs. Similarly, in our study, participants with a high resting motor threshold demonstrated changes in MEPs sizes. Moreover, biological variables including the asynchronous firing of motor units, variations in cortical volume and differences in skull thickness may contribute to the observed low stability of data both within and across subjects [125].

In conclusion, the absence of an exercise-induced effect on CMCT, MEPs amplitude and latency may indicate that the intervention protocol utilized does not significantly affect the structural integrity of the descending neural pathways. We postulate that our exercise protocol may have stronger effect on the resting motor threshold rather than CMCT. This inference is based on the notion that in-phase bilateral exercises predominantly engage transcallosal mechanisms [126, 127] and interhemispheric interactions [128].

### Exercise effects on resting motor threshold

A novel finding of this study is the bilateral decrease in resting motor threshold due to in-phase bilateral upper limb exercises. Considering that the resting motor threshold is a critical index of corticospinal excitability [114, 115] and a lower threshold suggests higher cortical excitability [129], our findings align with our initial hypothesis: in-phase bilateral exercises have a bilateral influence on the resting motor threshold.

In our study, three out of four participants exhibited a bilateral decrease in resting motor threshold. However, one participant showed a decrease only in the left (non-dominant) upper limb. This bilateral decrease suggests an enhancement in corticospinal plasticity [130, 131], which we attribute to our exercise regimen. Contrary to previous reports by Aramaki et al. [132] and Neva et al. [45], which found no change in resting motor threshold following antiphase bilateral exercises on the upper limbs in chronic stroke survivors, our data suggest that in-phase bilateral exercises can induce bilateral changes in individuals with RRMS.

Baseline measurements revealed a lower resting motor threshold in the right (dominant) upper limb compared to the left, corroborating findings from earlier studies [132, 133] in healthy individuals. Interestingly, a more pronounced decrease in the resting motor threshold was observed in the left (non-dominant) limb during our exercises, mirroring the effects reported by Waller et al. [127] in chronic stroke survivors, where the non-dominant limb experienced more significant improvements post-training. This suggests enhanced interhemispheric facilitation, particularly in the hemisphere corresponding to the non-dominant limb.

Previous research in chronic stroke survivors [43, 134] has indicated the role of transcallosal pathways in modulating bilateral cortical excitability. Activation of these pathways typically involves a concurrent decrease in interhemispheric inhibition and an increase in intracortical facilitation [42, 43, 45, 135]. Luft et al. [136] demonstrated that in-phase bilateral training activated cortical regions such as the ipsilesional precentral gyrus and contralesional superior frontal gyrus in stroke survivors, as shown by functional MRI. Additionally, Whitall et al. [44] highlighted the effectiveness of in-phase bilateral exercises for enhancing corticospinal plasticity in chronic stroke survivors. Our research extends these findings to individuals with RRMS, proposing that in-phase bilateral training can also enhance cortical plasticity and clinical outcomes in this population.

While aerobic training is known to improve corticospinal plasticity [137–140], it is essential to differentiate the effects of exercise intensity from those of the movement mechanism. To this end, we maintained constant HR below the aerobic threshold (70% of the maximum HR [109]) to ensure that our observations stemmed from the specific in-phase bilateral mechanism and not general exercise effects.

However, we observed high variability in the data for Participant D and the right limb of Participant A. This variability could relate to individual disease progression, as these participants are nearing a decade since the onset of RRMS—a point at which many transitions to a progressive stage [141, 142]. Such progression may account for the observed neural excitability changes [143, 144].

Lastly, our study design did not anticipate exercise-induced effects during the follow-up phase, as no exercise was prescribed. A descriptive analysis of this phase revealed a bilateral trend towards baseline levels, starting from the first month post-intervention. An increase in the resting motor threshold during follow-up—when exercise was absent—suggests a reversible effect on corticospinal plasticity [130] and underscores the potential benefit of our exercise protocol.

### Exercise effects on clinical measures

During the intervention phase, we observed improvements across all clinical metrics, while the follow-up phase was characterized by a regression to pre-intervention conditions, suggesting the absence of sustained benefits from the exercise protocol. Nonetheless, the temporary enhancements during the intervention highlight the potential efficacy of the exercise regimen on clinical outcomes for individuals with MS.

Continuous engagement in exercise is well-documented to ameliorate a range of clinical symptoms in MS patients [145–148]. The exercise protocol in this study, which emphasized bilateral upper limb movements, led to measurable enhancements in manual dexterity and limb strength, as evidenced by improved scores in the Action Research Arm Test and the Isometric Dynamometry test, respectively. This enhancement is indicative of improved upper limb function, which is beneficial across various MS subtypes [149].

Additionally, all participants exhibited improvements in balance and dynamic gait, as measured by the Mini Balance Evaluation Systems Test and the Six Spot Step Test. While the exercise regimen primarily involved the upper limbs, the observed benefits in balance and gait likely stemmed from the circuit training structure of the physical activity [150, 151], which inadvertently involved gait and balance practice through transitions between exercises.

Cognitive gains were also found, with participants showing increased information processing speed on the Symbol Digit Modalities Test. These results align with findings by Sandroff et al. [152], who demonstrated cognitive improvements in MS patients following diverse exercise protocols. Notably, our study recorded simultaneous enhancements in motor skills—including balance, gait, and hand dexterity—and cognitive speed, supporting literature that underscores the interdependence of cognitive functions and bimanual coordination [153–156] and their collective impact on physical disability risk in MS [157].

Visual analysis of the Modified Fatigue Impact Scale data revealed discernible changes in individual fatigue levels, encompassing physical, cognitive, and psychosocial domains, during both intervention and follow-up phases relative to baseline. These findings are consistent with prior research indicating that improved clinical status in MS is often correlated with heightened fatigue perception [158–160], substantiating the notion that clinical improvements in our participants could be associated with the observed alterations in fatigue levels.

Our study presents several methodological considerations. Firstly, the limited sample size, while sufficient for a pilot study aimed at generating initial data to guide future research, may affect the generalizability of our results. To mitigate this, we employed a concurrent multiple baseline design [56–58], which allows for systematic and individualized data collection at multiple time points, enhancing the robustness of our findings despite the sample size.

Secondly, our TMS assessments were performed using traditional methods without neuronavigation system. Although use of neuronavigation system can enhance the precision of stimulating consistent motor cortex areas [161, 162], the reproducibility and variability of TMS measures are not necessarily compromised by the absence of such technology [163]. To ensure the reliability of our TMS data, we systematically collected and analyzed data from 30 suprathreshold stimuli for each participant [164], implementing stringent criteria to calculate mean values for each data point, thereby maintaining the integrity of our TMS assessments.

Lastly, the APB was the selected muscle to assess corticospinal excitability of the upper extremities. This choice, while standard, does not encompass the activity of other muscles such as the biceps brachii, or the wrist flexors and extensors, which may be more directly engaged with our exercises and could therefore provide a more nuanced view of corticospinal excitability changes due to their distinct biomechanical contributions to upper limb movements.

While our results provide preliminary insights into the influence of exercise on corticospinal excitability, they contrast with those of Snow et al. [85], who questioned the clinical relevance of motor threshold in MS due to a lack of reported correlations with clinical outcomes. To reconcile these differing perspectives, subsequent research should replicate our exercise protocol with larger sample size of people with MS to establish the resting motor threshold as a crucial marker of corticospinal plasticity in this cohort.

Furthermore, considering the resting motor threshold’s potential role in predicting rehabilitation outcomes [129, 165], in-depth analyses comparing hemispheric data and their clinical correlations are warranted. This approach could yield significant insights into the interplay between neural plasticity and patient recovery. Discrepancies in the literature regarding the increased resting motor threshold in MS [166, 167] and its association with hemispheric dominance [29, 166, 167] also merit further exploration. Subsequent studies should aim to clarify the relationship between resting motor threshold, hand dominance and clinical metrics.

Expanding the methodological toolkit to include various quantitative neuromechanical assessments, such as gait analysis and isokinetic dynamometry, would provide a more comprehensive assessment of the rehabilitation progress. These methods could complement the resting motor threshold measurements to offer a multifaceted understanding of neuromechanical function.

Lastly, the cognitive component of motor skills, particularly bimanual dexterity, represents an understudied domain in MS research. Future investigations should consider the cognitive-dexterity nexus as a valuable parameter for assessing the complex relationship between cognition and motor function across different MS subtypes. This integrative approach could potentially inform more targeted and effective rehabilitation strategies for this patient population.

This pilot study represents an initial effort to investigate the impact of in-phase bilateral upper limb exercises on corticospinal plasticity in individuals with RRMS. To the best of our knowledge, it is the first of its kind to explore this area. The limited participant number—though typical of pilot studies—may underlie the absence of observed effects on CMCT, contrary to our initial hypothesis. Future research with expanded sample sizes are warranted to validate and potentially substantiate the influence of such exercises on CMCT in this population.

Notwithstanding these limitations, our study has provided compelling evidence that in-phase bilateral upper limb exercises can modulate the resting motor threshold, a measure known for its simplicity and reliability in assessing neural plasticity [130, 131, 168, 169]. The findings lay the groundwork for further exploration of the resting motor threshold as a prognostic tool for corticospinal plasticity in MS. Additionally, the observed improvements in various clinical measures endorse the potential of in-phase bilateral upper limb exercises as a viable rehabilitation approach for enhancing the clinical outcomes of those with RRMS.

Our study, therefore, adds valuable preliminary evidence to the field and sets the stage for larger, more definitive trials that could ultimately refine rehabilitation strategies for RRMS patients, emphasizing the importance of individualized exercise protocols that leverage corticospinal plasticity for clinical benefit.

## Supporting information

S1 Appendix Modified fatigue impact scale. (PDF)

S2 Appendix Results from CMCT, MEPs amplitude and latency. (DOCX)

S3 Appendix Clinical assessment results. (DOCX)

S4 Appendix SPIRIT checklist. (PDF)

S5 Appendix Study protocol. (PDF)
