# Supplementary material for: A 12-week in-phase bilateral upper limb exercise protocol promoted neuroplastic and clinical changes in people with relapsing remitting multiple sclerosis: A registered report randomized single-case concurrent multiple baseline study
Source: PLoS One. 2024 Oct 17;19(10):e0299611. doi: 10.1371/journal.pone.0299611 (PMC11486400; doi:10.1371/journal.pone.0299611)
Supplement: S2 Appendix — (DOCX) [file pone.0299611.s002.docx]

**Table 1. Individual CMCT data across all assessment points during baseline, intervention and follow-up.**

| Participant | A | B | C | D | E |
| --- | --- | --- | --- | --- | --- |
| Left APB | | | | | |
| Assessment point | **Baseline phase** | | | | |
| 1 | 8.7 | 9.9 | 9.7 | 18.7 | 7.8 |
| 2 | 8.8 | 8.4 | 11.4 | 17.7 | 10.8 |
| 3 | 10.9 | 8.5 | 11.4 | 14.8 | 7.4 |
| 4 | N/A | 8.7 | 21.3 | 8.9 | 11.1 |
| 5 | N/A | N/A | 11 | 17 | 8.1 |
| 6 | N/A | N/A | N/A | 7.9 | 5.7 |
| 7 | N/A | N/A | N/A | N/A | * |
| Mean | 9.5 | 8.9 | 13 | 14.2 | 8.5 |
| Assessment point | **Intervention phase** | | | | |
| 1 | 14.4 | 7.1 | 17.1 | 15.5 | 7.2 |
| 2 | 8.5 | 10.9 | 9.4 | 17.9 | 11.4 |
| 3 | 7.9 | 17 | 10.1 | 5 | 8.5 |
| 4 | 9.6 | 17.2 | 8.6 | 11.3 | Ex |
| 5 | 9.3 | 10.8 | 9.9 | 8.4 | 11.6 |
| Mean | 9.9 | 12.6 | 11 | 11.6 | 9.7 |
| Assessment point | **Follow-up phase** | | | | |
| 1 | 9.8 | 8.9 | 8 | 10.3 | 10.5 |
| 2 | 10.9 | 16.7 | 11.1 | 16.5 | 12.1 |
| 3 | 10.1 | 11 | 11.8 | 12.6 | * |
| Mean | 10.3 | 12.2 | 10.3 | 13.1 | 11.3 |
| Right APB | | | | | |
| Assessment point | **Baseline phase** | | | | |
| 1 | 8.5 | 8.7 | 10.6 | 9 | 8.8 |
| 2 | 7.8 | 7.5 | 14.7 | 8.6 | 13 |
| 3 | 7 | 19.1 | 11.4 | 13.2 | Ex |
| 4 | N/A | 10.4 | 11.2 | 15.5 | 8.3 |
| 5 | N/A | N/A | 12.5 | 8.3 | 8.5 |
| 6 | N/A | N/A | N/A | 17.3 | 8.1 |
| 7 | N/A | N/A | N/A | N/A | * |
| Mean | 7.7 | 11.4 | 12.1 | 12 | 9.3 |
| Assessment point | **Intervention phase** | | | | |
| 1 | Ex | 8 | 12.2 | 14.3 | 5.9 |
| 2 | 7.9 | 8.8 | 12 | 17.1 | 6.5 |
| 3 | 5.3 | 9.3 | 12.1 | 14.6 | 7.2 |
| 4 | 10.2 | 8.4 | 10.4 | 12 | 6.9 |
| 5 | 5.9 | 8 | 12.8 | 9.3 | 9 |
| Mean | 7.3 | 8.5 | 11.9 | 13.4 | 7.1 |
| Assessment point | **Follow-up phase** | | | | |
| 1 | 8.6 | 8.5 | 11.8 | 19.2 | 7.5 |
| 2 | 8.7 | 8.7 | 11.9 | 23.8 | 11.1 |
| 3 | 9.1 | 8.2 | 11.2 | 10.6 | * |
| Mean | 8.8 | 8.5 | 11.6 | 17.9 | 9.3 |

APB, Abductor Pollicis Brevis; N/A, Non-Applicable; Ex, Exclusion; Individual data of CMCT (ms) for each phase are presented in Table 1. The assessment points during baseline phase were recorded according to the study design (i.e., concurrent multiple baseline design across subjects). (*) refers to the assessment points which were missed. Participant E couldn’t perform the last TMS assessment of baseline and follow-up phases, on both upper limbs. Three data points (i.e., left APB, fourth intervention point of participant E; right APB, first intervention point of participant A; right APB, third baseline point of participant E) were excluded from the analysis, because they didn’t satisfy our criteria during data collection. Mean values of each phase were used for the analysis of the CMCT. Prior to averaging, we manually rejected TMS-elicited waveforms with MEPs peak to peak amplitudes less than 50mV and MEPs latencies higher or lower than two standard deviations; therefore, we averaged the values derived from the remaining trials. By doing this, we ensure data quality and reliability.

**Table 2. Individual MEPs amplitude data across all assessment points during baseline, intervention and follow-up.**

| Participant | A | B | C | D | E |
| --- | --- | --- | --- | --- | --- |
| Left APB | | | | | |
| Assessment point | **Baseline phase** | | | | |
| 1 | 0.9 | 0.05 | 0.05 | 0.07 | 0.2 |
| 2 | 0.2 | 0.1 | 0.2 | 0.02 | 0.2 |
| 3 | 0.5 | 0.05 | 0.2 | 0.05 | 0.3 |
| 4 | N/A | 0.1 | 0.07 | 0.06 | 0.1 |
| 5 | N/A | N/A | 0.1 | 0.08 | 0.2 |
| 6 | N/A | N/A | N/A | 0.2 | 0.2 |
| 7 | N/A | N/A | N/A | N/A | * |
| Mean | 0.5 | 0.08 | 0.1 | 0.08 | 0.2 |
| Assessment point | **Intervention phase** | | | | |
| 1 | 0.9 | 0.05 | 0.05 | 0.06 | 0.1 |
| 2 | 0.1 | 0.1 | 0.2 | 0.06 | 0.1 |
| 3 | 0.06 | 0.06 | 0.08 | 0.1 | 0.09 |
| 4 | 0.2 | 0.07 | 0.1 | 0.2 | 0.05 |
| 5 | 0.01 | 0.06 | 0.07 | 0.3 | 0.08 |
| Mean | 0.1 | 0.07 | 0.1 | 0.1 | 0.09 |
| Assessment point | **Follow-up phase** | | | | |
| 1 | 0.3 | 0.05 | 0.09 | 0.06 | 0.1 |
| 2 | 0.3 | 0.05 | 0.1 | 0.05 | 0.08 |
| 3 | 0.07 | 0.08 | 0.1 | 0.08 | * |
| Mean | 0.2 | 0.06 | 0.1 | 0.06 | 0.09 |
| Right APB | | | | | |
| Assessment point | **Baseline phase** | | | | |
| 1 | 0.8 | 0.06 | 0.5 | 0.09 | 0.2 |
| 2 | 0.3 | 0.1 | 0.08 | 0.08 | 0.07 |
| 3 | 0.2 | 0.1 | 0.2 | 0.05 | 0.06 |
| 4 | N/A | 0.05 | 0.2 | 0.06 | 0.2 |
| 5 | N/A | N/A | 0.1 | 0.2 | 0.2 |
| 6 | N/A | N/A | N/A | 0.1 | 0.2 |
| 7 | N/A | N/A | N/A | N/A | * |
| Mean | 0.47 | 0.09 | 0.2 | 0.1 | 0.1 |
| Assessment point | **Intervention phase** | | | | |
| 1 | 0.06 | 0.08 | 0.1 | 0.08 | 0.1 |
| 2 | 0.06 | 0.1 | 0.1 | 0.05 | 0.2 |
| 3 | 0.06 | 0.06 | 0.1 | 0.07 | 0.1 |
| 4 | 0.3 | 0.3 | 0.1 | 0.05 | 0.1 |
| 5 | 0.2 | 0.09 | 0.1 | 0.07 | 0.2 |
| Mean | 0.1 | 0.1 | 0.1 | 0.07 | 0.1 |
| Assessment point | **Follow-up phase** | | | | |
| 1 | 0.2 | 0.1 | 0.2 | 0.05 | 0.2 |
| 2 | 0.6 | 0.09 | 0.3 | 0.05 | 0.06 |
| 3 | 0.1 | 0.1 | 0.07 | 0.09 | * |
| Mean | 0.3 | 0.1 | 0.2 | 0.06 | 0.1 |

APB, Abductor Pollicis Brevis; N/A, Non-Applicable; Individual data of MEPs amplitude (mV) for each phase are presented in Table 2. The assessment points during baseline phase were collected according to the study design (i.e., concurrent multiple baseline design across subjects). (*) refers to the assessment points which were missed. Participant E couldn’t perform the last TMS assessment of baseline and follow-up phases, on both upper limbs. Mean values of each phase were used for the analysis of the MEPs amplitude. Prior to averaging, we manually rejected TMS-elicited waveforms with MEPs peak to peak amplitudes less than 50mV and MEPs latencies higher or lower than two standard deviations; therefore, we averaged the values derived from the remaining trials. By doing this, we ensure data quality and reliability. Throughout the within and between-subjects’ visual analysis, data variability and instability could be observed to all participants, during all study phases and for both upper limbs.

**Table 3. Individual MEPs latency data across all assessment points during baseline, intervention and follow-up.**

| Participant | A | B | C | D | E |
| --- | --- | --- | --- | --- | --- |
| Left APB | | | | | |
| Assessment point | **Baseline phase** | | | | |
| 1 | 24.2 | 22.8 | 23.2 | 34.3 | 23.5 |
| 2 | 25 | 21.3 | 24.3 | 32.2 | 23.4 |
| 3 | 26.2 | 21.3 | 24.9 | 31.3 | 23.6 |
| 4 | N/A | 21.7 | 35.4 | 25.5 | 26 |
| 5 | N/A | N/A | 24.2 | 30.4 | 23.4 |
| 6 | N/A | N/A | N/A | 23.9 | 21.4 |
| 7 | N/A | N/A | N/A | N/A | * |
| Mean | 25.1 | 21.8 | 26.4 | 29.6 | 23.5 |
| Assessment point | **Intervention phase** | | | | |
| 1 | 30.7 | 20.1 | 30.3 | 30.2 | 22.4 |
| 2 | 24.5 | 23.8 | 22.3 | 34.4 | 25.5 |
| 3 | 23 | 29.8 | 23.6 | 18.6 | 23 |
| 4 | 24.6 | 28.2 | 23.8 | 26.2 | 15.5 |
| 5 | 25.1 | 23.5 | 22.8 | 23.5 | 26.5 |
| Mean | 25.6 | 25.1 | 24.5 | 26.6 | 22.6 |
| Assessment point | **Follow-up phase** | | | | |
| 1 | 24.9 | 22.4 | 21.3 | 25.5 | 22.9 |
| 2 | 24.7 | 28.1 | 23.2 | 28.5 | 25.5 |
| 3 | 24.4 | 24.7 | 25.4 | 25.9 | * |
| Mean | 24.6 | 25 | 23.3 | 26.6 | 24.2 |
| Right APB | | | | | |
| Assessment point | **Baseline phase** | | | | |
| 1 | 24.3 | 21.9 | 24.5 | 24.7 | 22.5 |
| 2 | 23.6 | 20.5 | 28.1 | 23.9 | 27 |
| 3 | 22.8 | 32.2 | 24.5 | 28.8 | 18.8 |
| 4 | N/A | 23.7 | 24.3 | 29 | 22.9 |
| 5 | N/A | N/A | 24.2 | 21.5 | 23.7 |
| 6 | N/A | N/A | N/A | 31.9 | 22.3 |
| 7 | N/A | N/A | N/A | N/A | * |
| Mean | 23.6 | 24.6 | 25.1 | 26.6 | 22.9 |
| Assessment point | **Intervention phase** | | | | |
| 1 | 18.8 | 21.1 | 25.1 | 29.5 | 20.5 |
| 2 | 23.8 | 21.8 | 24.7 | 30.4 | 21.3 |
| 3 | 20.7 | 22.5 | 25.4 | 29.2 | 21.5 |
| 4 | 25.7 | 21.5 | 23.4 | 26.7 | 21.7 |
| 5 | 19.8 | 21.6 | 25.9 | 24.1 | 23.3 |
| Mean | 21.7 | 21.7 | 24.9 | 28 | 21.6 |
| Assessment point | **Follow-up phase** | | | | |
| 1 | 23.8 | 21.9 | 24.6 | 32.4 | 22 |
| 2 | 24.2 | 20.8 | 24.6 | 37.9 | 25 |
| 3 | 24.8 | 22.1 | 24.6 | 23.8 | * |
| Mean | 24.3 | 21.6 | 24.6 | 31.3 | 23.5 |

APB, Abductor Pollicis Brevis; N/A, Non-Applicable; Individual data of MEPs latency (ms) for each phase are presented in Table 3. The assessment points during baseline phase were collected according to the study design (i.e., concurrent multiple baseline design across subjects). (*) refers to the assessment points which were missed. Participant E couldn’t perform the last TMS assessment of baseline and follow-up phases, on both upper limbs. Mean values of each phase were used for the analysis of the MEPs latency. Prior to averaging, we manually rejected TMS-elicited waveforms with MEPs peak to peak amplitudes less than 50mV and MEPs latencies higher or lower than two standard deviations; therefore, we averaged the values derived from the remaining trials. By doing this, we ensure data quality and reliability. Throughout the within and between-subjects’ visual analysis, data variability and instability could be observed to all participants, during all study phases and for both upper limbs.
