# Supplementary material for: A 12-week in-phase bilateral upper limb exercise protocol promoted neuroplastic and clinical changes in people with relapsing remitting multiple sclerosis: A registered report randomized single-case concurrent multiple baseline study
Source: PLoS One. 2024 Oct 17;19(10):e0299611. doi: 10.1371/journal.pone.0299611 (PMC11486400; doi:10.1371/journal.pone.0299611)
Supplement: S3 Appendix — (DOCX) [file pone.0299611.s003.docx]

**Table 1. Individual clinical measures data across all assessment points during baseline, intervention and follow-up.**

| Participant | A | B | C | D | E |
| --- | --- | --- | --- | --- | --- |
| Mini Balance Evaluation Test (maximum score = 28) | | | | | |
| Baseline phase | 22 | 23 | 19 | 21.4 | 20 |
| Intervention phase | 27 | 24.8 | 21.4 | 24.6 | 25 |
| Follow-up phase | 26.3 | 23.3 | 18.3 | 21.6 | 22.3 |
| NAP /  standard error | 1  / 0.06 | 1  /0.04 | 1  /0.03 | 1  /0.02 | 1  /0.02 |
| p-value < | 0.05 | 0.01 | 0.01 | 0.01 | 0.01 |
| Six Spot Step Test | | | | | |
| Baseline phase | 7 | 10.4 | 11 | 7.8 | 9.5 |
| Intervention phase | 5.6 | 8.5 | 9.8 | 6.4 | 7.5 |
| Follow-up phase | 5.6 | 8.6 | 10.4 | 6.6 | 7.6 |
| NAP /  standard error | 1  / 0.06 | 1  /0.04 | 1  /0.03 | 1  /0.02 | 1  /0.02 |
| p-value < | 0.05 | 0.01 | 0.01 | 0.01 | 0.01 |
| Symbol Digit Modalities Test | | | | | |
| Baseline phase | 73.6 | 69.5 | 71.2 | 51.7 | 54.5 |
| Intervention phase | 79.8 | 73.6 | 79.8 | 65.6 | 63.2 |
| Follow-up phase | 76 | 71 | 72 | 60.6 | 56.3 |
| NAP /  standard error | 1  / 0.06 | 1  /0.04 | 1  /0.03 | 1  /0.02 | 1  /0.02 |
| p-value < | 0.05 | 0.01 | 0.01 | 0.01 | 0.01 |

NAP, Non Overlap of All Pairs; Mean scores and statistical analysis of the clinical measures are presented in Table 1. In the statistical analysis, the values of NAP and *p*-value refer to the results from the comparison between baseline and intervention phase only. The follow-up phase was not included in the statistical analysis. The Mini Balance Evaluation Test assesses the dynamic balance, functional mobility and gait, which showed a significant improvement to all participants during the intervention phase. However, during the follow-up phase there was a reduction in the performance of all participants and return to the baseline values as were expected, since there was no exercise during that phase. The Six Spot Step Test which examines the gait, showed significant improvement to all participants during the intervention phase and lasted across the follow-up phase as well. The Symbol Digit Modalities Test which assess the information processing speed, showed significant improvement to all participants during the intervention phase, with a tendency to return to the baseline level during the follow-up phase.

**Table 2. Individual Action Research Arm Test data across all assessment points during baseline, intervention and follow-up.**

| Participant | A | B | C | D | E |
| --- | --- | --- | --- | --- | --- |
| Left Upper Limb (maximum score = 57) | | | | | |
| Baseline phase | 8.7 | 9.6 | 9.8 | 11.4 | 13.4 |
| Intervention phase | 6.6 | 8 | 8.8 | 8 | 8.7 |
| Follow-up phase | 6.7 | 8.1 | 9.7 | 9.6 | 10.6 |
| NAP /  standard error | 1  / 0.06 | 1  /0.04 | 1  /0.03 | 1  /0.02 | 1  /0.02 |
| p-value < | 0.05 | 0.01 | 0.01 | 0.01 | 0.01 |
| Right Upper Limb (maximum score = 57) | | | | | |
| Baseline phase | 8 | 9 | 10.1 | 10.3 | 12.2 |
| Intervention phase | 5.8 | 7.9 | 8.4 | 8.7 | 8.4 |
| Follow-up phase | 6.6 | 8 | 8.8 | 9.5 | 9.2 |
| NAP /  standard error | 1  / 0.06 | 1  /0.04 | 1  /0.03 | 1  /0.02 | 1  /0.02 |
| p-value < | 0.05 | 0.01 | 0.01 | 0.01 | 0.01 |

NAP, Non Overlap of All Pairs; Mean scores and statistical analysis of the Action Research Arm Test, for both upper limbs and for each participant, are presented in Table 2. In the statistical analysis the values of NAP and *p*-value refer to the results from the comparison between baseline and interventions phase only. A significant improvement was observed to all participants, in both upper limbs, during the intervention phase. However, during the follow-up phase there was a reduction for both left and right upper limbs in the performance (i.e., increased values) of all participants, which return to the baseline values as were expected, since there was no exercise.

**Table 3. Individual Isometric Dynamometer Test data across all assessment points during baseline, intervention and follow-up.**

| Participant | A | | B | | C | | D | | E | |
| --- | --- | --- | --- | --- | --- | --- | --- | --- | --- | --- |
|  | **L** | **R** | **L** | **R** | **L** | **R** | **L** | **R** | **L** | **R** |
| Shoulder Flexors | | | | | | | | | | |
| Baseline phase | 10.1 | 12.3 | 10.3 | 11.9 | 10.9 | 12 | 12.2 | 13.3 | 10.7 | 12.1 |
| Intervention phase | 15.8 | 19.3 | 16 | 19.4 | 13.1 | 14.5 | 14.4 | 17.6 | 14.8 | 15.4 |
| Follow-up phase | 13.4 | 16.2 | 14.2 | 17.6 | 12 | 12.7 | 12.3 | 15.7 | 13.5 | 12.9 |
| NAP /  standard error | 1  /0.06 | 1  /0.06 | 1  /0.04 | 1  /0.04 | 1  /0.03 | 1  /0.03 | 1  /0.02 | 1  /0.02 | 1  /0.02 | 1  /0.02 |
| p-value < | 0.05 | 0.05 | 0.01 | 0.01 | 0.01 | 0.01 | 0.01 | 0.01 | 0.01 | 0.01 |
| Shoulder Extensors | | | | | | | | | | |
| Baseline phase | 15.3 | 15.1 | 15 | 16.1 | 12.6 | 11.9 | 15.6 | 15.6 | 13.1 | 14.8 |
| Intervention phase | 20.8 | 21.3 | 20.9 | 21.6 | 14.5 | 15.2 | 19.2 | 20 | 17 | 17.3 |
| Follow-up phase | 18.1 | 16.7 | 19.1 | 20 | 13.3 | 13.9 | 18.1 | 18.1 | 15.7 | 16.5 |
| NAP /  standard error | 1  /0.06 | 1  /0.06 | 1  /0.04 | 1  /0.04 | 1  /0.03 | 1  /0.03 | 1  /0.02 | 1  /0.02 | 1  /0.02 | 1  /0.02 |
| p-value < | 0.05 | 0.05 | 0.01 | 0.01 | 0.01 | 0.01 | 0.01 | 0.01 | 0.01 | 0.01 |
| Shoulder Internal Rotators | | | | | | | | | | |
| Baseline phase | 8.6 | 13.4 | 10.3 | 11.4 | 8.9 | 10.1 | 11.4 | 14.4 | 8 | 12.5 |
| Intervention phase | 13.2 | 16.8 | 13.9 | 16.8 | 12.6 | 12.7 | 14.7 | 18.2 | 12.9 | 18.2 |
| Follow-up phase | 10.8 | 15.2 | 12.8 | 14.6 | 11.5 | 12.1 | 13.1 | 17.2 | 11.4 | 16.9 |
| NAP /  standard error | 1  /0.06 | 1  /0.06 | 1  /0.04 | 1  /0.04 | 1  /0.03 | 1  /0.03 | 1  /0.02 | 1  /0.02 | 1  /0.02 | 1  /0.02 |
| p-value < | 0.05 | 0.05 | 0.01 | 0.01 | 0.01 | 0.01 | 0.01 | 0.01 | 0.01 | 0.01 |
| Shoulder External Rotators | | | | | | | | | | |
| Baseline phase | 9.1 | 9.2 | 11.4 | 11.3 | 10 | 10.5 | 11.5 | 12.3 | 9.9 | 11.5 |
| Intervention phase | 12 | 12.2 | 12.7 | 13.1 | 12.4 | 13.2 | 15.6 | 14.7 | 12.5 | 13.7 |
| Follow-up phase | 11.4 | 11.9 | 11.3 | 11.6 | 11.4 | 12 | 14.8 | 13.1 | 11.5 | 12 |
| NAP /  standard error | 1  /0.06 | 1  /0.06 | 1  /0.04 | 1  /0.04 | 1  /0.03 | 1  /0.03 | 1  /0.02 | 1  /0.02 | 1  /0.02 | 1  /0.02 |
| p-value < | 0.05 | 0.05 | 0.01 | 0.01 | 0.01 | 0.01 | 0.01 | 0.01 | 0.01 | 0.01 |
| Shoulder Adductors | | | | | | | | | | |
| Baseline phase | 13.5 | 13.2 | 12.6 | 13.7 | 10.3 | 12.1 | 17.1 | 17.2 | 12.2 | 12.3 |
| Intervention phase | 17.9 | 20.1 | 18.2 | 20.1 | 12.6 | 14.4 | 21.4 | 21.4 | 19.7 | 21.5 |
| Follow-up phase | 17.2 | 18.7 | 16.3 | 18.9 | 11 | 13 | 19.4 | 19.5 | 17.8 | 16.8 |
| NAP /  standard error | 1  /0.06 | 1  /0.06 | 1  /0.04 | 1  /0.04 | 1  /0.03 | 1  /0.03 | 1  /0.02 | 1  /0.02 | 1  /0.02 | 1  /0.02 |
| p-value < | 0.05 | 0.05 | 0.01 | 0.01 | 0.01 | 0.01 | 0.01 | 0.01 | 0.01 | 0.01 |
| Shoulder Abductors | | | | | | | | | | |
| Baseline phase | 10 | 12.6 | 11.2 | 13.9 | 10.1 | 11.3 | 11.1 | 13.2 | 12.3 | 13.7 |
| Intervention phase | 16.7 | 18.8 | 17 | 19 | 13.2 | 12.8 | 17 | 17.8 | 17.3 | 16.8 |
| Follow-up phase | 15.1 | 15 | 16.1 | 17.8 | 12.3 | 11 | 15.8 | 16.2 | 14.5 | 14.3 |
| NAP /  standard error | 1  /0.06 | 1  /0.06 | 1  /0.04 | 1  /0.04 | 1  /0.03 | 1  /0.03 | 1  /0.02 | 1  /0.02 | 1  /0.02 | 1  /0.02 |
| p-value < | 0.05 | 0.05 | 0.01 | 0.01 | 0.01 | 0.01 | 0.01 | 0.01 | 0.01 | 0.01 |
| Shoulder Horizontal Adductors | | | | | | | | | | |
| Baseline phase | 9.5 | 9.1 | 10.4 | 10.3 | 9.6 | 9.3 | 11.4 | 12 | 9.2 | 11.5 |
| Intervention phase | 12.7 | 13.3 | 13 | 13.4 | 11.1 | 12.7 | 14 | 13.8 | 12.5 | 15.2 |
| Follow-up phase | 11 | 9.8 | 11.2 | 12.4 | 10.8 | 11.3 | 12.3 | 12.5 | 10.9 | 13.3 |
| NAP /  standard error | 1  /0.06 | 1  /0.06 | 1  /0.04 | 1  /0.04 | 1  /0.03 | 1  /0.03 | 1  /0.02 | 1  /0.02 | 1  /0.02 | 1  /0.02 |
| p-value < | 0.05 | 0.05 | 0.01 | 0.01 | 0.01 | 0.01 | 0.01 | 0.01 | 0.01 | 0.01 |
| Shoulder Horizontal Abductors | | | | | | | | | | |
| Baseline phase | 13.6 | 14 | 12.2 | 12.4 | 9.6 | 10.3 | 13.4 | 14.2 | 9.4 | 12.2 |
| Intervention phase | 17.3 | 19.4 | 17.6 | 19.6 | 11.3 | 13.3 | 16.1 | 19.9 | 12.4 | 14.6 |
| Follow-up phase | 15.5 | 17.2 | 16.4 | 18.2 | 10.4 | 11.8 | 14.5 | 19.1 | 10.5 | 12.9 |
| NAP /  standard error | 1  /0.06 | 1  /0.06 | 1  /0.04 | 1  /0.04 | 1  /0.03 | 1  /0.03 | 1  /0.02 | 1  /0.02 | 1  /0.02 | 1  /0.02 |
| p-value < | 0.05 | 0.05 | 0.01 | 0.01 | 0.01 | 0.01 | 0.01 | 0.01 | 0.01 | 0.01 |
| Elbow flexors | | | | | | | | | | |
| Baseline phase | 15.2 | 14.5 | 12 | 16.7 | 12 | 12.5 | 12.3 | 14.2 | 12.1 | 15.2 |
| Intervention phase | 21.6 | 23 | 22.1 | 23 | 16.6 | 18.3 | 17.2 | 17.3 | 18.4 | 22.3 |
| Follow-up phase | 18.9 | 15.5 | 20.8 | 21.6 | 15.6 | 16.7 | 14.8 | 14.4 | 16.3 | 18.6 |
| NAP /  standard error | 1  /0.06 | 1  /0.06 | 1  /0.04 | 1  /0.04 | 1  /0.03 | 1  /0.03 | 1  /0.02 | 1  /0.02 | 1  /0.02 | 1  /0.02 |
| p-value < | 0.05 | 0.05 | 0.01 | 0.01 | 0.01 | 0.01 | 0.01 | 0.01 | 0.01 | 0.01 |
| Elbow extensors | | | | | | | | | | |
| Baseline phase | 11 | 12.2 | 10.5 | 12.3 | 9.4 | 9.8 | 11.5 | 12.8 | 10.2 | 12 |
| Intervention phase | 17.7 | 21.8 | 17.3 | 21.5 | 11.7 | 13.5 | 15.4 | 17.4 | 17.5 | 17.2 |
| Follow-up phase | 11.9 | 13.3 | 15.3 | 19.1 | 10.2 | 12.2 | 14.3 | 16.1 | 15 | 15.3 |
| NAP /  standard error | 1  /0.06 | 1  /0.06 | 1  /0.04 | 1  /0.04 | 1  /0.03 | 1  /0.03 | 1  /0.02 | 1  /0.02 | 1  /0.02 | 1  /0.02 |
| p-value < | 0.05 | 0.05 | 0.01 | 0.01 | 0.01 | 0.01 | 0.01 | 0.01 | 0.01 | 0.01 |
| Hand Grip | | | | | | | | | | |
| Baseline phase | 18.4 | 20.7 | 10.3 | 10.3 | 12.8 | 16.4 | 20.9 | 25.5 | 15.5 | 18.1 |
| Intervention phase | 23.5 | 25.1 | 23.6 | 25.4 | 15.3 | 18.6 | 25.4 | 28.3 | 20.5 | 25.4 |
| Follow-up phase | 20.8 | 23.8 | 22.4 | 23.4 | 14.1 | 17.7 | 23.8 | 25.9 | 16.5 | 23.1 |
| NAP /  standard error | 1  /0.06 | 1  /0.06 | 1  /0.04 | 1  /0.04 | 1  /0.03 | 1  /0.03 | 1  /0.03 | 1  /0.02 | 1  /0.02 | 1  /0.02 |
| p-value < | 0.05 | 0.05 | 0.01 | 0.01 | 0.01 | 0.01 | 0.01 | 0.01 | 0.01 | 0.01 |

L, Left Upper Limb; R, Right Upper Limb; NAP, Non Overlap of All Pairs; Mean scores (expressed in Kg) and statistical analysis of the Dynamometer test for each participant. All participants showed significant improvement for both left and right upper limbs, during the intervention phase. During the follow-up phase there was a reduction for both left and right upper limbs in the performance of all participants, which return to the baseline values as we were expected, since there was no exercise. In the statistical analysis the values of NAP and *p*-value refer to the results from the comparison between the data of the baseline and the intervention phase only.

**Table 4.** **Individual Modified Fatigue Impact Scale data across all assessment points during baseline, intervention, and follow-up.**

| Participant | A | B | C | D | E |
| --- | --- | --- | --- | --- | --- |
| Baseline phase | 43 | 53 | 45 | 45 | 44 |
| Intervention phase | 15 | 41 | 20 | 20 | 30 |
| Follow-up phase_1 | 18 | 46 | 29 | 29 | 31 |
| Follow-up phase_2 | 18 | 50 | 32 | 32 | 34 |
| Follow-up phase_3 | 19 | 52 | 34 | 34 | 35 |

Mean scores of the Modified Fatigue Impact Scale. All patients reported an improvement (i.e., decrease of values) regarding their fatigue level. However, after the intervention phase all participants, except the participant B, reported that their fatigue level still was improved. Participant B returned (i.e., increase of values) to its baseline level.
