# Supplementary material for: A 12-week in-phase bilateral upper limb exercise protocol promoted neuroplastic and clinical changes in people with relapsing remitting multiple sclerosis: A registered report randomized single-case concurrent multiple baseline study
Source: PLoS One. 2024 Oct 17;19(10):e0299611. doi: 10.1371/journal.pone.0299611 (PMC11486400; doi:10.1371/journal.pone.0299611)
Supplement: S4 Appendix — (PDF) [file pone.0299611.s004.pdf]

Appendix 4_ SPIRIT Checklist: Recommended items to address in a clinical trial protocol and related documents*

Section/item	Item No	Description
**Administrative information**		
Title	1	Descriptive title identifying the study design, population, interventions, and, if applicable, trial acronym<br>Investigation of in-phase bilateral exercise effects on corticospinal plasticity in relapsing remitting multiple sclerosis: a registered report single-case concurrent multiple baseline design across five subjects, NCT05367947
Trial registration	2a	Trial identifier and registry name. If not yet registered, name of intended registry<br>ClinicalTrials.gov NCT05367947
	2b	All items from the World Health Organization Trial Registration Data Set<br>- Primary registry and trial identifying number: ClinicalTrials.gov NCT05367947<br>- Date of registration in primary registry: 10 May, 2022<br>- Secondary Identifying numbers: N/A<br>- Source(s) of monetary or material support: Cyprus University of Technology<br>- Primary sponsor: Cyprus University of Technology<br>- Secondary sponsor(s): The Cyprus Institute of Neurology and Genetics, The Cyprus Foundation for Muscular Dystrophy Research<br>- Contact for public queries: DS; sokratous.physio@gmail.com; Cyprus University of Technology; Vragadinou 15, Limassol, 3041; 00357 25002294<br>- Contact for Scientific Queries: DS, NK, Cyprus University of Technology.<br>- Public title: In-phase Bilateral Exercises in People With Relapsing Remitting Multiple Sclerosis<br>- Scientific title: Investigation of in-phase bilateral exercise effects on corticospinal plasticity in relapsing-remitting multiple sclerosis

- Countries of recruitment: Cyprus

- Health condition(s) or problem(s) studied: exercise, clinical condition, corticospinal plasticity

- Intervention(s): In-phase bilateral exercise of the upper limbs in five people with relapsing remitting multiple sclerosis.

- Key Inclusion and Exclusion Criteria:

Ages eligible for study: 30-70 years; Sexes eligible for study: both

Inclusion criteria: Patients with relapsing-remitting multiple sclerosis, Expanded Disability Status Scale score between three and five

Exclusion criteria: Metal implants, history of cardiovascular or any disease affecting the central nervous system other than multiple sclerosis, pregnancy, epileptic seizures, Spasticity level on upper or lower limbs more than 1+ (slight increase in muscle tone) according to Modified Ashworth Scale.

- Study type:	Interventional
Estimated Enrollment :	5 participants
Allocation:	N/A
Intervention Model:	Single Group Assignment
Intervention Model Description:	The study follows a concurrent case studies. The specific desig phases
Masking:	None (Open Label)
Masking Description:	Participants are people with mul<br>Investigators are health professi
Primary Purpose:	Basic Science
- Date of first enrolment:	N/A
- Sample size:	5
- Recruitment status of this trial:	Pending, participants are not yet being recruited or enrolled at any site
- Primary Outcome(s):	Central Motor Conduction Time [Time Frame: Through study completion, an average 35 weeks]

		- Key secondary outcomes:	Resting Motor Threshold, Motor Evoked Potential Amplitude and Latency, Mini Balance Evaluation Systems Test,<br>Six Spot Step Test,<br>Action Research Arm Test,<br>Isometric Dynamometer,<br>Symbol Digit Modalities Test,<br>Modified Fatigue Impact Scale<br>[Time Frame for all measures: Through study completion, an average 35 weeks].
		- Ethics Review:	Status: Registration No. ΕΕΒΚ ΕΠ 2022 32<br>Date of approval: pending<br>Name and contact details: Cyprus National Bioethics Committee; 22 Laertou Str., 2365 Ayios Dometios, Nicosia, (00357) 22-809038 / 22-809039 / cnbc@bioethics.gov.cy
		- Completion date:	July 2021 is the estimated study completion date.
		- IPD sharing statement:	Undecided
Protocol version	3	Date and version identifier<br>Issue date: 10 May, 2022<br>Authors: D.S., C.C.C., E.Z.P., K.M. and N.K.	
Funding	4	Sources and types of financial, material, and other support<br>Equipment, consumables and diagnostic devices have been provided by The Cyprus Institute of Neurology and Genetics.	
Roles and responsibilities	5a	Names, affiliations, and roles of protocol contributors<br>D.S. [Department of Rehabilitation Sciences, Faculty of Health Sciences, Cyprus University of Technology, Limassol, Cyprus; Physiotherapy Unit, Neurology Clinics, The Cyprus Institute of Neurology and Genetics, Nicosia, Cyprus].<br>C.C.C. [Centre for Neuroscience and Integrative Brain Research (CENIBRE), University of Nicosia Medical School, Nicosia, Cyprus].<br>E.Z.P. [Neuroepidemiology Department, The Cyprus Institute of Neurology and Genetics, Nicosia, Cyprus].	

N.K. [Department of Rehabilitation Sciences, Faculty of Health Sciences, Cyprus University of Technology, Limassol, Cyprus].
K.M. [Biostatistics Unit, The Cyprus Institute of Neurology and Genetics, Nicosia, Cyprus].

Author contribution
D.S., C.C.C., E.Z.P., and N.K., are responsible for the conception and the experimental design.
D.S., and K.M., are responsible to collect, analyse and interpret the data.
D.S., and N.K., are responsible to draft the manuscript.
N.K., C.C.C., and K.M., revised the manuscript critically for important intellectual content.

5b Name and contact information for the trial sponsor

Investigator Name: Mr. Dimitris Sokratous

Investigator Official Title: MSc

Investigator Affiliation: Cyprus University of Technology, Department of Rehabilitation Sciences, Cyprus University of Technology. Vragadinou 15, Limassol, 3041, 00357 25002294

5c Role of study sponsor and funders, if any, in study design; collection, management, analysis, and interpretation of data; writing of the report; and the decision to submit the report for publication, including whether they will have ultimate authority over any of these activities

N/A

5d Composition, roles, and responsibilities of the coordinating centre, steering committee, endpoint adjudication committee, data management team, and other individuals or groups overseeing the trial, if applicable (see Item 21a for data monitoring committee)

Principal investigator (PI)
Design and revision of the protocol
Organising steering committee meetings
Managing CTO [clinical trials office]
Publication of study reports
Members of TMC [Trial Management Committee]

Steering committee
(PI, research consultant, senior neurologist, senior physiotherapist)

Agreement of final protocol
All lead investigators will be steering committee members.
Recruitment of patients and liaising with principle [sic] investigator
Reviewing progress of study and if necessary agreeing changes to the protocol and/or facilitate the smooth running of the study.

Trial management committee
(PI, research physician, senior physiotherapist, administrator)
Study planning
Organisation of steering committee meetings
Responsible for trial master file
Advice for lead investigators
Data verification
Randomisation

Data manager committee
(senior physiotherapist, biostastician)
Maintenance of trial IT system and data entry
Data verification
Analysis plan
Data collection and completion

Lead investigators
Senior neurologist and senior physiotherapist are responsible for identification, recruitment.
Lead investigators will be steering committee members.

Introduction

Background and rationale	6a	Description of research question and justification for undertaking the trial, including summary of relevant studies (published and unpublished) examining benefits and harms for each intervention

Introduction: Relapsing Remitting MS (RRMS) is the most common type of MS which is characterised by periods of relapses, that generate unexpected motor symptoms in both body sides.

Mechanism: Motor symptoms are associated with the corticospinal tract integrity, which is quantified by means of corticospinal plasticity which can be probed via transcranial magnetic stimulation (TMS). Several factors, such as exercise and interlimb coordination, can influence corticospinal plasticity.

Existing knowledge: Previous work in healthy and in stroke patients showed that the greatest improvement in corticospinal plasticity occurred during in-bilateral arm exercises [1–4]. Despite the broad literature on the effects of different types of exercises on the neuroplasticity in people with RRMS [5–8], it is unclear whether in-phase bilateral exercises can promote motor related neuroplastic changes in people with MS (pwMS).

Need for a trial: In light of evidence that pwMS have bilateral cortical lesions which cause bilateral changes of corticospinal tract integrity, these findings raise the question about the effects of bilateral exercises on corticospinal plasticity. Such effects would provide strong evidence about whether exercise, in particular in-bilateral exercise, can influence the corticospinal plasticity in people with RRMS.

6b Explanation for choice of comparators

According to the MS guidelines, various types of exercises are recommended for promoting neuroplasticity and improvement of clinical symptoms. Therefore, a within, as well as a between cases analysis and comparison can be used to identify the possible effects. Alteration in corticospinal plasticity and changes in clinical symptoms of the participants during the three experimental phases (i.e., baseline, intervention, follow-up) will encourage the effectiveness of the proposed intervention.

Objectives 7 Specific objectives or hypotheses

7.1. Research hypothesis

In-phase bilateral exercises of the upper limbs, improve corticospinal plasticity and clinical condition of people with RRMS.

7.2. Primary objectives

To determine if the exercises which include the two types (i.e., in-phase; anti-phase) of in-phase bilateral movement for the upper limbs can promote corticospinal plasticity of people with RRMS.

7.3. Secondary objectives

To determine if the exercises which include the two types (i.e., in-phase; anti-phase) of in-phase bilateral movement for the upper limbs can improve the clinical condition (i.e., motor skills; cognitive functions) of people with RRMS.

Trial design	8	Description of trial design including type of trial (eg, parallel group, crossover, factorial, single group), allocation ratio, and framework (eg, superiority, equivalence, noninferiority, exploratory)<br><br>The study follows a concurrent multiple baseline design across subjects, without blinding and has been designed according to the 'What Works Clearinghouse' criteria for single case studies [9]. According to Kratochwill et al. [9], three participants, with collection of three data points for each across different phases is the minimum number needed to meet the standard criteria, while four or more is recognized as more reliable. Therefore, we aim to include five participants to ensure the reliability of the results in case of dropouts, as well as to record several data points across the baseline phase, five data points during the intervention phase and three data points in the follow up phase. However, there will be a randomization of the order in which the participants will be allocated. During the experimental procedure, all participants will begin the study with the baseline phase at the same time while the intervention phase is introduced staggered across patients and time. The intervention will be introduced systematically in one patient while baseline data collection continues in the others without any intervention.

Methods: Participants, interventions, and outcomes

Study setting	9	Description of study settings (eg, community clinic, academic hospital) and list of countries where data will be collected. Reference to where list of study sites can be obtained<br><br>The entire study, including intervention and assessment of outcome measures, will be conducted in the neurophysiology lab and in the physiotherapy unit of The Cyprus Institute of Neurology and Genetics (CING).
Eligibility criteria	10	Inclusion and exclusion criteria for participants. If applicable, eligibility criteria for study centres and individuals who will perform the interventions (eg, surgeons, psychotherapists)<br><br>All participants will be recruited and evaluated by the senior neurologist of CING. Moreover, all participants will read and sign a written informed consent while all procedures are approved and conducted in accordance with the ethical guidelines of the Cyprus National Bioethics Committee before recruitment (see Appendix 1_Informed Consent Form).<br><br>*10.1. Inclusion criteria*<br>1) diagnosed with RRMS,<br>2) Expanded Disability Status Scale score between three and five (58),<br>3) aged between 30 and 70 years,<br>4) no relapse within 30 days,

5) Mini Mental State of Examination score between 24 and 30 (no cognitive impairment) (59).

10.2. Exclusion criteria
1) metal implants,
2) history of any disease affecting the central nervous system other than MS,
3) history of cardiovascular disease,
4) mental disorders,
5) severe orthopaedic disorders,
6) pregnancy,
7) visual deficit,
8) hearing impairments,
9) epileptic seizures,
10) spasticity level on upper or lower limbs more than 1+ (slight increase in muscle tone) according to Modified Ashworth Scale (60).

Interventions	11a	Interventions for each group with sufficient detail to allow replication, including how and when they will be administered

According to the study design (i.e., multiple baseline design across subjects [9], the intervention will be introduced systematically in one patient while baseline data collection continues in the others without any intervention. During the experimental procedure, all participants will begin the study with the baseline phase at the same time while the intervention phase is introduced staggered across patients and time. Therefore, five people with RRMS will be allocated randomly. The intervention protocol will last for 12 consecutive weeks (30-60 minutes /session x 3 sessions/week) and include in-phase bilateral movements of the upper limbs, adapted to different sports activities and to functional training. Specifically, each session will consist of one to three sets, consisting of 10–15 repetitions of 9 different exercises targeting large muscle groups of the upper limbs (shoulder flexors, extensors, rotators, abductors and adductors, elbow flexors and extensors, hand and finger flexors and extensors). Additionally, three exercises will target large lower limb muscle groups (hip flexors, extensors, abductors and adductors, knee and ankle flexors and extensors) to be performed in between the upper limbs exercises to allow relaxation of the upper limb muscles.

The specific exercises will include sports activities of basic technical skills of basketball (e.g., different types of passing, catching and throwing the ball) and volleyball (e.g., different types of passing and receiving the ball), whereas the fitness exercises will include shoulder rows, shoulder lateral raises, elbow flexions, elbow extensions, using resistance elastic bands [7], as well as exercises with the patients' own body weight (e.g., pushups, TRX) [10]. To maintain the interest of the participants, the exercise program will be modified throughout the course of the 12-week intervention period via changing the level of difficulty. For example,

elastic bands with different resistance levels will be used, the number of repetitions and sets will vary along with the specific exercise and body position (e.g., from sitting to standing). Every intervention session will consist of a five minutes' warm-up (i.e., whole body range of motion exercises), followed by the main sport activities and fitness exercise protocol as described above, and a cool down session for five minutes (i.e., passive stretching exercises of the muscle groups which are involved in the main part).

11b Criteria for discontinuing or modifying allocated interventions for a given trial participant (eg, drug dose change in response to harms, participant request, or improving/worsening disease)

Fatigue

In order to maintain individual fatigue, each exercise protocol will be adapted to the individual needs and with sufficient resting time, as well as continues monitoring.

However, each participant has to complete at least 27 (75%) out of 36 sessions in order to be included in the data analysis.

11c Strategies to improve adherence to intervention protocols, and any procedures for monitoring adherence (eg, drug tablet return, laboratory tests)

Adherence information sessions

Face-to-face adherence information sessions for all participants will take part before starting the experimental procedures. These sessions will include:

- The importance of following study guidelines and instructions about the type of exercises and the specific assessment procedures.

- Importance of calling nurses or/and doctors if experiencing any problems or symptoms possibly related to the study.

- There will be brief discussion of reasons for feeling any unexpected symptoms (e.g., pain, fatigue)

Moreover, participants will have an opportunity to ask questions from the initial session will be reviewed as needed.

Adherence assessments

To enhance validity of data, an individual electronic data form will be used to record all neurophysiological and clinical assessments, which will be stored in a secure study computer

	11d	Relevant concomitant care and interventions that are permitted or prohibited during the trial<br><br>Participants will be advised to continue their usual prescribed medication throughout the study duration, and they will be advised to continue their usual daily routine avoiding receiving any other exercise program during the study.
Outcomes	12	Primary, secondary, and other outcomes, including the specific measurement variable (eg, systolic blood pressure), analysis metric (eg, change from baseline, final value, time to event), method of aggregation (eg, median, proportion), and time point for each outcome. Explanation of the clinical relevance of chosen efficacy and harm outcomes is strongly recommended<br><br>**Primary Outcome Measures**<br><br>Using electromyography (EMG) signals, we will analyse bilateral cortical excitability and bilateral central motor conduction time to determine corticospinal plasticity and therefore to test the primary hypothesis [3,11].<br><br>**Secondary Outcome Measures**<br><br>We will investigate the effects of the specific exercises protocol on the resting motor threshold, on the motor evoke potential (MEP) amplitude and latency of Abductor Pollicis Brevis muscle, which will define cortical excitability [3,12].Clinical symptoms using clinical assessment (i.e., gait, balance, strength, hand dexterity, cognitive functions) Mini Balance Evaluation Systems Test measures dynamic balance, functional mobility, and gait in neurological patients, including people with RRMS [13], the Six Spot Step Test is an assessment tool that evaluates a complex range of sensorimotor functions, part of which are lower limb strength, spasticity, coordination, as well as balance [14]. We well assess the isometric muscle force of major muscle groups with the use of the muscle controller (Kinvent Biomechanique, Montpelier, France) which is a dynamometer used in the evaluation and rehabilitation of muscle strength that provides real time biofeedback [15]. Also, we will employ the oral form of the Symbol Digit Modalities Test which assesses the f information processing speed [16]. Finally, we will perform the Modified Fatigue Impact Scale which is a short questionnaire that requires the participants to describe the effects of fatigue during the past four weeks [17].

Participant timeline	13	Time schedule of enrolment, interventions (including any run-ins and washouts), assessments, and visits for participants. A schematic diagram is highly recommended (see Figure)

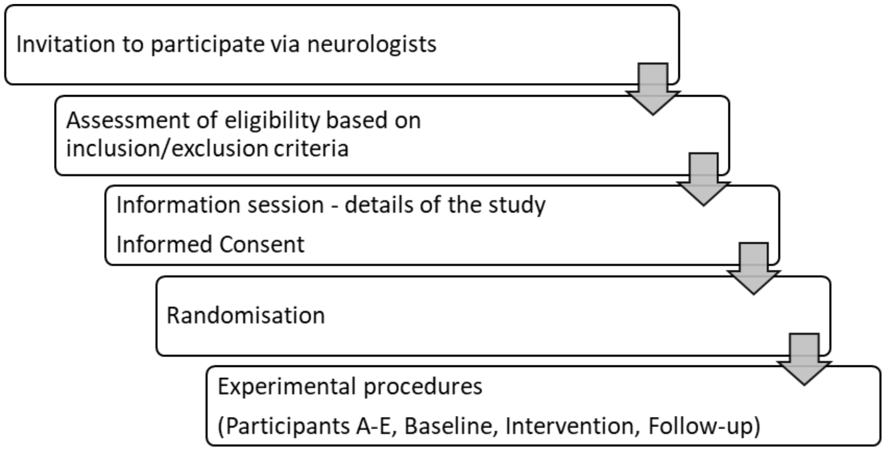

For the experimental procedures see also Appendix 3_ Figure 1.

Sample size	14	Estimated number of participants needed to achieve study objectives and how it was determined, including clinical and statistical assumptions supporting any sample size calculations

The study follows a concurrent multiple baseline design across subjects, without blinding and has been designed according to the 'What Works Clearinghouse' criteria for single case studies [9]. According to Kratochwill et al. [9], three participants, with collection of three data points across different phases is the minimum number needed to meet the standard criteria, while four or more is recognized as more reliable. We aim to include five participants to ensure the reliability of the results in case of dropouts, as well as to record several data points across the baseline phase, five data points during the intervention phase and three data points in the follow up phase. During the experimental procedure, all participants will begin the study with the baseline phase at the same time while the intervention phase is introduced staggered across patients and time (see Appendix 2). The intervention will be introduced systematically in one patient while baseline data collection continues in the others without any intervention. The cause-effect inference can be clearly verified by the staggered duration through separate baseline phases [18]. Subsequently, if the intervention (i.e., in-phase bilateral exercises) is the sole cause of improvement in participants' conditions, the proposed outcome measures will not change for the participants that remain in the baseline phase but will be improved only for those in the intervention phase.

Recruitment	15	Strategies for achieving adequate participant enrolment to reach target sample size<br><br>One of the affiliated organizations is the Cyprus Institute of Neurology and Genetics (CING) which is a medical and biomedical translation center, promoting patient care, research and educational programs on neurological disease, including MS. Therefore, patients will be recruited mainly from the senior neurologist of CING throughout the patient registry (database). Once identified in the database, patients<br><br>potentially eligible for the specific study will be contacted by the senior neurologist who will explain the study and ascertains the patient's<br><br>interest. If interested, the patients will be examined in the clinical laboratories where more detailed evaluations will be performed, so to confirm that fulfil the study criteria.

Methods: Assignment of interventions (for controlled trials)

Allocation:

Sequence generation	16a	Method of generating the allocation sequence (eg, computer-generated random numbers), and list of any factors for stratification. To reduce predictability of a random sequence, details of any planned restriction (eg, blocking) should be provided in a separate document that is unavailable to those who enrol participants or assign interventions<br><br>The participants will be randomly assigned in numerical order from 1 to 5. So the one who will be placed in number 1 will start the exercise protocol first and the one in number 5 will be last with a difference of one week with each other.
Allocation concealment mechanism	16b	Mechanism of implementing the allocation sequence (eg, central telephone; sequentially numbered, opaque, sealed envelopes), describing any steps to conceal the sequence until interventions are assigned<br><br>Participants will be allocated by generate random numbers using the ECXEL, after all participants will assign inform consent. After that all participants will start from the baseline assessments. The intervention will be introduced systematically in one patient, week by week, while baseline data collection continues in the others without any intervention.

Implementation	16c	Who will generate the allocation sequence, who will enrol participants, and who will assign participants to interventions<br><br>All patients who will fulfil the inclusion criteria will be randomized and then informed about the study procedures, so finally to give consent for participation. Randomisation will be requested by the senior neurologist responsible for recruitment according inclusion/exclusion criteria from the CING. Then the list of participants` randomised order will be send to the main researcher (i.e., senior physiotherapist of CING) who will coordinate the study. The therapist will give the information about the exercise protocol and the related neurophysiological and clinical assessments.
Blinding (masking)	17a	Who will be blinded after assignment to interventions (eg, trial participants, care providers, outcome assessors, data analysts), and how<br><br>Due to the nature of the study, which is a concurrent multiple baseline design across subjects [9], neither participants nor staff can be blinded to allocation. A certified fitness instructor designed the exercise protocols and two experienced physiotherapists will perform all adequate assessments who will feed all data into the computer separate datasheets so that the researchers can proceed with the analysis plan.
	17b	If blinded, circumstances under which unblinding is permissible, and procedure for revealing a participant's allocated intervention during the trial<br><br>Due to the nature of the study, which is a concurrent multiple baseline design across subjects [9], neither participants nor staff can be blinded.

Methods: Data collection, management, and analysis

Data collection methods	18a	Plans for assessment and collection of outcome, baseline, and other trial data, including any related processes to promote data quality (eg, duplicate measurements, training of assessors) and a description of study instruments (eg, questionnaires, laboratory tests) along with their reliability and validity, if known. Reference to where data collection forms can be found, if not in the protocol<br><br>**Primary Outcome Measures**<br><br>Using electromyography (EMG) signals, we will analyse bilateral cortical excitability and bilateral central motor conduction time to determine corticospinal plasticity and therefore to test the primary hypothesis [3,11].

Secondary Outcome Measures

We will investigate the effects of the specific exercises protocol on the resting motor threshold and on the motor evoke potential (MEP) amplitude and latency of Abductor Pollicis Brevis muscle, which will define cortical excitability, while we will use the MEP latency to calculate the central motor conduction time [3,12]. Clinical symptoms using clinical assessment (i.e., gait, balance, strength, hand dexterity, cognitive function) Mini Balance Evaluation Systems Test measures dynamic balance, functional mobility, and gait in neurological patients, including people with RRMS [13], the Six Spot Step Test is an assessment tool that evaluates a complex range of sensorimotor functions, part of which are lower limb strength, spasticity, coordination, as well as balance [14]. We well assess the isometric muscle force of major muscle groups with the use of the muscle controller (Kinvent Biomechanique, Montpelier, France) which is a dynamometer used in the evaluation and rehabilitation of muscle strength that provides real time biofeedback [15]. Also, we will employ the oral form of the Symbol Digit Modalities Test which assesses the f information processing speed [16]. Finally, we will perform the Modified Fatigue Impact Scale which is a short questionnaire that requires the participants to describe the effects of fatigue during the past four weeks [17].

Quality control

All scientific researchers are experienced and specialized in motor and cognitive functions in healthy individuals but also in patients with neurological and psychiatric disorders. Also, the two main organizations that will work together to carry out this study are specialized in the field of rehabilitation of patients with chronic neurological diseases. The first institution is the Cyprus University of Technology (CUT) and specifically the Department of Rehabilitation Sciences of the School of Health Sciences, has a well-equipped rehabilitation clinic in which systematic studies are carried out for the rehabilitation of neurological patients. The second institution, which is the CING, is one of the most recognized centers for providing health services in Cyprus to people with chronic neurological diseases. The staff of the Neurophysiology laboratory and the Physiotherapy Unit of the CING, are specialized in evaluation and treatment services to people with chronic neurological diseases such as MS.

As depicted in the experimental procedures scheme (see Appendix 3_Figure 1), all patients will begin the baseline phase simultaneously and during this phase, each participant will be assessed on primary and secondary outcome measures by two physiotherapists who are staff members of the CING. In order to ensure quality of the intervention a certified fitness instructor designed the protocols in collaboration with the

senior physiotherapist of the CING. During the intervention phase we will perform several neurophysiological (see primary outcome measures) and five clinical assessments (see secondary outcome measures) (i.e., once a week), to collect several data for every participant. Additionally, every participant will undergo three follow-up assessments in total, after finishing the exercise protocol, to explore possible long-lasting effects. Each follow-up assessment includes both primary and secondary outcome measures, performed by the two experienced physiotherapists.

18b

Plans to promote participant retention and complete follow-up, including list of any outcome data to be collected for participants who discontinue or deviate from intervention protocols

Due to our study design, several data can be collected from the beginning of the participants` allocation. Several clinical and neurophysiological assessment will be recorded for each participant. However, participants` will have the opportunity to be systematically informed about their own clinical condition as well as the level of performance during the intervention phase. Another important factor which will promote participants` retention is the opportunity they have to exercise in a different way that they used to, as well as they offer to them a scientific exercise program under supervision without any cost.

Data management 19

Plans for data entry, coding, security, and storage, including any related processes to promote data quality (eg, double data entry; range checks for data values). Reference to where details of data management procedures can be found, if not in the protocol

All information related to the specific program, including data for all participants, will be collected by the principal investigator and the senior physiotherapist of the CING who participating in the research study. From the moment each participant is enrolled to the research study he / she receives a participant ID number (e.g., 101, 102). From this point on, participant's name will not be used again and the participant ID will be used. Only the principal investigator and the senior physiotherapist of the CING will have access to the name of the participants. Five years after the data collection, all the material stored electronically will be permanently deleted by the principal investigator. In addition, copies of the collected data (questionnaires, clinical and cognitive tests, forms, etc.) will be immediately destroyed.

All data will be stored in the office of the principal investigator and the senior physiotherapist of the CING. All computers that will be used in this research study for data storage will be offline. In addition, logging in to computers requires an account password that will be known only to the two previously mentioned researchers of the study. Finally, the buildings are also protected by a security company during working hours.

Statistical methods	20a	Statistical methods for analysing primary and secondary outcomes. Reference to where other details of the statistical analysis plan can be found, if not in the protocol

To investigate possible effects of our protocol we will follow recommended guidelines [19], in which we will perform a separate analysis for each of the outcome measures, in all experimental phases (i.e., baseline, intervention and follow up). We will perform a visual analysis first, in order to determine whether there is a functional relationship between the intervention and the outcome measures, and secondly, we will perform a quantitative analysis method to evaluate the magnitude of the intervention effect, provided there is evidence from the visual analyses [19]. We will perform all neurophysiological and clinical assessments to each participant according to the number of data points during each phase (i.e., baseline, intervention, follow up).

Initially, a visual analysis will be conducted and presented graphically in a spaghetti plot, in order to define whether there is a functional relation between the intervention and the outcome measures (54). During the visual analysis, six features of the research design graphed data will be examined: level, trend, stability, immediacy of the effect, overlap, and consistency. Over the within-phase examination an evaluation of level, trend and stability will be examined. Level will be reported from the mean score of each dependent variable and trend will determine whether the data points are monotonically decreased or increased. Stability will be estimated based on the percentage of data points falling within 15% of the phase median, if this is higher than 80% then we assume that this criterion is met. Additionally, over the between-phase examination an evaluation of overlapping data among baseline and intervention phases, consistency of data patterns and immediacy of effect will be performed.

Secondly, in order to estimate the individual-level effect sizes, we will use three different methods of quantitative analysis, as suggested by ‘What Works Clearinghouse’ [9], the standardized mean difference (Cohen’s d), the standardized mean difference with correction for small sample sizes (Hedges’ g) and piecewise regression analysis which does not only reflect the immediate intervention effect, but also the intervention effect across time. Multilevel modelling, which is recommended by the ‘What Works Clearinghouse’ and the single case educational design, specific mean difference index will be used to estimate the magnitude of the effect across cases and compared to the effect obtained by the single level estimates. All tests will be two sided and statistical analysis will be performed using the statistical software R (https://www.r-project.org/).

	20b	Methods for any additional analyses (eg, subgroup and adjusted analyses)<br><br>N/A due to the study design.
	20c	Definition of analysis population relating to protocol non-adherence (eg, as randomised analysis), and any statistical methods to handle missing data (eg, multiple imputation)<br><br>N/A due to the study design.
**Methods: Monitoring**		
Data monitoring	21a	Composition of data monitoring committee (DMC); summary of its role and reporting structure; statement of whether it is independent from the sponsor and competing interests; and reference to where further details about its charter can be found, if not in the protocol. Alternatively, an explanation of why a DMC is not needed<br><br>A data monitoring committee is not needed due to the study design, although a biostastician, who is independent and blinded to the study procedures will perform all statistical analysis. All data sheets which are completed throughout the assessments of the primary and secondary outcome measures will be given to her in order to proceed with the adequate statistical analysis.
	21b	Description of any interim analyses and stopping guidelines, including who will have access to these interim results and make the final decision to terminate the trial<br><br>Due to the study design, an independent and blinded to the study procedures biostatistician will perform the analysis of the data at the end of each phase (i.e., baseline, intervention, follow up). The principle investigator will have access to the results but without any possibility to terminate the trial until all participants complete the intervention phase.
Harms	22	Plans for collecting, assessing, reporting, and managing solicited and spontaneously reported adverse events and other unintended effects of trial interventions or trial conduct<br><br>An adverse event will be defined as any unpleasant medical occurrence in a subject. Adverse events will be collected after the subject has provided consent and enrolled in the study. If a subject experiences an adverse event after the informed consent document is signed but the subject has not started to perform any intervention, the event will be reported as not related to the study. All adverse events occurring during the intervention will be recorded and reported to the senior neurologist. A

		serious adverse event for this study is any untoward medical occurrence that is believed by the investigators to be causally related to study intervention and results in any of the following: Life-threatening condition (that is, immediate risk of death); severe or permanent disability, musculoskeletal pain, fatigue. Serious adverse events occurring after a subject is discontinued from the study will NOT be reported unless the investigators feels that the event may have been caused by the exercise protocol or any of the study procedures. Investigators will determine relatedness of an event to study intervention, as well as whether the event is unexpected or unexplained given the subject's clinical course and previous clinical conditions. Due to the study design, participants will be clinically examined systematically, thus there will be a systematic monitoring so any possible adverse event will be reported.
Auditing	23	Frequency and procedures for auditing trial conduct, if any, and whether the process will be independent from investigators and the sponsor<br><br>The PI and the steering committee, who are members of the entire research team, will frequently (i.e., once a week) audit the overall quality and completeness of the data, examine source documents and confirm that all health professionals who are included in the study have complied with the requirements of the protocol. Also, they will review all source documents as needed, whether data sheets are completed and be updated.
**Ethics and dissemination**		
Research ethics approval	24	Plans for seeking research ethics committee/institutional review board (REC/IRB) approval<br><br>This protocol and the template informed consent forms contained in Appendix 3 will be reviewed and approved by the Cyprus National Bioethics Committee with respect to scientific content and compliance with applicable research and human subjects' regulations. The protocol, site-specific informed consent forms (local language and English versions), participant education and recruitment materials, and other requested documents also will be reviewed and approved by the ethical review bodies. The PI will make safety and progress reports to the Cyprus National Bioethics Committee at least annually and within three months of study termination or completion.

Protocol amendments	25	Plans for communicating important protocol modifications (eg, changes to eligibility criteria, outcomes, analyses) to relevant parties (eg, investigators, REC/IRBs, trial participants, trial registries, journals, regulators)<br><br>Any modifications to the protocol which may impact on the conduct of the study, potential benefit of the patient or may affect patient safety, including changes of study objectives, study design, patient population, sample sizes, study procedures, or significant administrative aspects will approved by the Cyprus National Bioethics Committee prior to implementation.
Consent or assent	26a	Who will obtain informed consent or assent from potential trial participants or authorised surrogates, and how (see Item 32)<br><br>The PI and the senior physiotherapist of the CING will introduce the study procedures to the participants. Participants will also receive information sheets and then will be able to provide written consent from since are adults without any cognitive impairment. All information sheets and consent form are written in local language which is Greek).
	26b	Additional consent provisions for collection and use of participant data and biological specimens in ancillary studies, if applicable<br><br>N/A
Confidentiality	27	How personal information about potential and enrolled participants will be collected, shared, and maintained in order to protect confidentiality before, during, and after the trial<br><br>Participants' study information will not be released outside of the study without the written permission of the participant. Only the PI and the senior physiotherapist of the CING will have access to the name of the participants. Five years after the data collection, all the material stored electronically will be permanently deleted by the principal investigator. In addition, copies of the collected data (questionnaires, clinical and cognitive tests, forms, etc.) will be immediately destroyed.<br>All data will be stored in the office of the principal investigator and the senior physiotherapist of the CING. All computers that will be used in this research study for data storage will be offline. In addition, logging in to computers requires an account password that will be known only to the two previously mentioned researchers of the study. Finally, the buildings are also protected by a security company during working hours.
Declaration of interests	28	Financial and other competing interests for principal investigators for the overall trial and each study site<br><br>PI declares no conflict of interest.

Access to data	29	Statement of who will have access to the final trial dataset, and disclosure of contractual agreements that limit such access for investigators<br><br>The steering group will have access to the full trial dataset in order to ensure the overall results. To ensure confidentiality, data dispersed to project team members will be blinded of any identifying participant information.
Ancillary and post-trial care	30	Provisions, if any, for ancillary and post-trial care, and for compensation to those who suffer harm from trial participation<br><br>Patients who will be enrolled into the study are covered by through the standard General Health System.
Dissemination policy	31a	Plans for investigators and sponsor to communicate trial results to participants, healthcare professionals, the public, and other relevant groups (eg, via publication, reporting in results databases, or other data sharing arrangements), including any publication restrictions<br><br>The scientific integrity of the project requires that all the data from all participants will be analysed and reported to all team members after completion of the experimental procedures.<br><br>Each paper or abstract must be reviewed or/and approved by the steering committee, before they will be submitted to an appropriate scientific journal or/and a scientific conference.<br><br>The entire research study may terminate at the planned target of 1,5 years after the last participant will be enrolled to the intervention phase. Regardless of the timing and circumstances of the end of the study, close-out will proceed in two stages:<br><br>- The first stage is the interim period for analysis and documentation of study results.<br><br>- The second stage is the debriefing of participants and dissemination of study results, in which the paper with the final results will be submitted to an appropriate journal. We expect to take about 5 to 6 months, after the last participant will finish the follow up phase, to compile the final paper.

	31b	Authorship eligibility guidelines and any intended use of professional writers<br><br>PI, steering and data manager committee will be the lead authors of the entire research study. If some protocol authors are not named authors of subsequent publications, their role in protocol design will be acknowledged in the published report.
	31c	Plans, if any, for granting public access to the full protocol, participant-level dataset, and statistical code<br><br>No later than 2 years after the data collection and analysis, we will deliver a completely data set to an appropriate data archive for sharing purposes, including published and unpublished analysis.
**Appendices**		
Informed consent materials	32	Model consent form and other related documentation given to participants and authorised surrogates<br>Appendix 2a_ Informed Consent Form<br>Appendix 2b_ Information sheet of TMS
Biological specimens	33	Plans for collection, laboratory evaluation, and storage of biological specimens for genetic or molecular analysis in the current trial and for future use in ancillary studies, if applicable<br>N/A

*It is strongly recommended that this checklist be read in conjunction with the SPIRIT 2013 Explanation & Elaboration for important clarification on the items. Amendments to the protocol should be tracked and dated. The SPIRIT checklist is copyrighted by the SPIRIT Group under the Creative Commons "Attribution-NonCommercial-NoDerivs 3.0 Unported" license.

Appendix 2a_ Informed Consent Form

**CONSENT FORMS**<br>**for in a research program**<br>(The forms are comprised of 8 pages)
**Title of the Programme you are invited to participate**
Investigation of in-phase bilateral exercise effects on corticospinal plasticity in relapsing remitting multiple sclerosis.

This form provides the explanations in plain and comprehensible language regarding what is being requested from you and/or what will happen to you if you agree to join the program:

1. All risks that may exist or any inconvenience you may incur from participating in the program.
2. The person(s) who will have access to your information and will arise from the program you will take part in and/or other material/data that you voluntarily provide for the program.
3. The time period during which the Principal Investigator will have access to your information and/or material concerning you.
4. What the Principal Investigator hope to learn as a result of your participation.
5. Estimation of the benefit that can be gained for researchers and/or sponsors of this program.
6. **You should not participate if you do not wish to, or if you have any concerns about your participation in the program.**
7. If you decide to join, you must indicate if you have participated in any other research programs within the last 12 months.
8. If you decide not to participate and you are a patient, your treatment will not be affected by your decision.
9. **You are free to withdraw your consent to participating in the programme at any time.**
10. If you are a patient, your decision to withdraw your consent will not have any effect on your treatment.
11. All pages of consent forms must bear your full name and signature.

Principal Investigator of the Program you are invited to participate in
Dr. Nikos Konstantinou, Assistant Professor, Department of Rehabilitation Sciences, Cyprus University of Technology. Vragadinou 15, Limassol, 3041, telephone number: 00357 25002294, email: nikos.konstantinou@cut.ac.cy

Surname:	....	Name:	....
Signature:	....	Date:	....

CONSENT FORMS

for in a research program

(The forms are comprised of 8 pages)

**Title of the Programme you are invited to participate**
Investigation of in-phase bilateral exercise effects on corticospinal plasticity in relapsing remitting multiple sclerosis.

Program Duration:
5 years

Do you give consent for yourself or for someone else?	
If you have responded for another person, please provide details and name.	
Question	YES or NO
Did you fill in your consent forms personally?	
Over the past 12 months, have you been involved in any other research program?	
Did you read and understand the information regarding patients and/or volunteers?	
Have you had the opportunity to ask questions and discuss the Program?	
Have you been given satisfactory answers and explanations to any of your questions?	
Do you understand that you can withdraw from the programme whenever you wish?	
Do you understand that if you withdraw, you do not need to give any explanations for your decision?	
(For patients) do you understand that, if you withdraw, there will be no impact on any treatment you get or you can get in the future?	
**Do you agree to join the program?**	
With whom did you speak with?	

Surname:	………………………………………………………	Name:	…………………………………………
Signature:	………………………………………………………	Date:	…………………………………………

CONSENT FORMS

for in a research program

(The forms are comprised of 8 pages)

**Title of the Programme you are invited to participate**
Investigation of in-phase bilateral exercise effects on corticospinal plasticity in relapsing remitting multiple sclerosis.

Brief description of the program (procedures and purpose).
You are invited to participate in a research of the Department of Rehabilitation Sciences of the Cyprus University of Technology (CUT) in collaboration with the Cyprus Institute of Neurology and Genetics (CING). Before deciding whether or not to participate, it is important to understand the main goal of this research study. Take some time to carefully read the information below, as well as you can discuss it with others if you wish. Moreover, you can ask our team if there is anything that is not clear or you do not understand or if you would like more information about this information sheet or consent form. Take time to decide whether or not you want to participate.<br>The main goal of this research study, is to investigate the effects on the clinical condition and quality of life in patients with MS, throughout a program of different types of exercises (i.e., in-phase bilateral exercises), which are adapted to different sports activities and fitness exercises. The study is expected to be an important tool in the implementation of future treatment programs in patients with MS. Participation in this research is voluntary. You are not expected to receive any immediate financial or personal benefit; however, your participation will greatly contribute to the development of science.<br>**Risks of participation**: There are no risks and no complications from your participation in this study.<br>**Confidentiality**: The data collected is anonymous and you do not need to provide any information about your identity. No information received will be able to identify you.<br>**Research participation procedure**: The duration of this exercise program is 12 weeks for each participant who will take part in three weekly sessions (45-60 minutes / session). Furthermore, before the beginning of the intervention, during the 12 weeks of the intervention and in one year after the end of the intervention, there will be frequent clinical assessments of motor and cognitive functions, neurophysiological examinations (corticospinal plasticity), as well as recording of results from questionnaires concerning the quality of life and fatigue for each participant.<br>**Right of non-participation or withdrawal**: Your participation is completely voluntary and you should participate only if you wish. Choosing not to participate or leave during the research program will not have a negative effect on you, will not cost you anything and will not affect any other treatment you may receive.

Surname:	....	Name:	....
Signature:	....	Date:	....

CONSENT FORMS

for in a research program

(The forms are comprised of 8 pages)

**Title of the Programme you are invited to participate**
Investigation of in-phase bilateral exercise effects on corticospinal plasticity in relapsing remitting multiple sclerosis.

Details of what will be requested and/or what will happen to program participants			
If you agree to participate in this research study, we will ask you to come to CING. This research study includes frequent assessments before, during and after the end of a 12-week program based on sports and functional training exercises. More specifically, in the Physiotherapy Unit of ING, frequent clinical assessments and the sessions of the specific exercises will be performed based on protocol. Also, in the Neurophysiology lab of CING will be performed the neurophysiological assessments with the use of Transcranial Magnetic Stimulation (TMS). During each clinical assessment you will be asked to perform specific and very reliable motor tests, such as balance, strength, gait and functionality of the upper limbs. You will also be asked to complete a cognitive skills test for the evaluation of the information processing speed. It is important to mention that between each test you will be given enough time to rest so as to avoid fatigue. Specialist physiotherapists will record your results in each test and at the end of the program if you wish you can be given all the findings. Thru each neurophysiological assessment, measurements will be recorded in relation to the corticospinal plasticity of the two cerebral hemispheres and the central motor conduction time of the upper limbs via TMS. At this point we will ask you to sit comfortably in an armchair in order to activate the motor cortex of the brain. Using special electrodes that will be placed in both hands, we will monitor the activity of a muscle in each hand respectively. When applying TMS, we will activate your brain cells with simple magnetic pulses produced by an insulated coil which we will place on your scalp. Each pulse travels through your scalp, causing a small electric current in the cortex (the outer part of the brain). The goal is to find the area of the brain that corresponds to the specific muscle of the hand in which we have placed the special electrode. It is important to know that magnetic pulses can cause a slight tingling sensation on your scalp. This sensation is usually not unpleasant but sometimes it can actually cause an annoying sensation.<br>The exercise program in which you will be invited to take part, includes exercises that contain in-phase bilateral movements and are adapted to different sports activities and functional exercises. All exercises will be under the guidance and supervision of specialized sport scientist and physiotherapists experienced in the field of neurorehabilitation and you will need to wear the appropriate sportswear.<br>The purpose of this research study is to investigate the effect of specific exercises on your clinical condition as well as on the functioning of the central nervous system. It is important to know that you can request to stop and leave this program at any time without any excuse and with no consequences.			
Surname:	…………………………………………………………	Name:	………………………………………
Signature:	…………………………………………………………	Date:	………………………………………

CONSENT FORMS

for in a research program

(The forms are comprised of 8 pages)

**Title of the Programme you are invited to participate**
Investigation of in-phase bilateral exercise effects on corticospinal plasticity in relapsing remitting multiple sclerosis.

Details of any risks that may exist or any inconvenience that program participants may incur
By providing a series of exercises for a long time, you may feel tired. In case this happens you will be given specific guidelines in order to recover. All researchers are health professionals and experienced in the field of neurorehabilitation and exercising in chronic diseases.

Details of what information and/or what material will be collected under the program, who will have access to it and for how long.
You will not be asked for any personal data that could lead to your identification. During the program, all data will be collected in relation to your motor and cognitive condition, as well as your motor cortex activity via TMS. At the same time, you will be given the Safety Check Form for TMS, which you will be asked to state if you have a history of specific diseases.

Surname:	………………………………………………………	Name:	………………………………………
Signature:	………………………………………………………	Date:	………………………………………

CONSENT FORMS

for in a research program

(The forms are comprised of 8 pages)

Title of the Programme you are invited to participate
Investigation of in-phase bilateral exercise effects on corticospinal plasticity in relapsing remitting multiple sclerosis.

WHERE APPLICABLE, FUTURE STORAGE AND USE OF BIOLOGICAL SAMPLES AND PERSONAL DATA:<br>Please note and sign either left or right	
Except for the purposes of this program that will last for ... . years<br><br>**I consent**: ☐<br>Signature:	Except for the purposes of this study that will last for ... . years<br><br>**I do not consent**: ☐<br>Signature:
that my biological samples (buccal swabs, saliva or DNA) and genetic information which shall be stored at the ………………………………………………. **may be kept for more than ……… years and be used in future studies** upon authorization of the Cyprus National Bioethics Committee (CNBC), following the relevant application for renewal by the Principal Investigator of this Program, I understand that matters of confidentiality will always be in force.	

If new information that directly affects your health is discovered, would you like to be informed?		
YES ☐	NO ☐	I CANNOT MAKE A DECISION NOW. PLEASE ASK AGAIN IF NEEDED ☐

Details of what data will be generated for you within the program, who will have access to them and for how long.
All data that will be gained, are related to motor and cognitive skills, as well as to the motor cortex activity. Only the research team will have access to this data and it will be destroyed after 5 years. In case of publication of the results of the present study in a scientific journal or in any other conference, any of your personal data will not be published and you will not be able to identify yourself with any published material.

Surname:	………………………………………………………	Name:	………………………………………
Signature:	………………………………………………………	Date:	………………………………………

CONSENT FORMS

for in a research program

(The forms are comprised of 8 pages)

Title of the Programme you are invited to participate
Investigation of in-phase bilateral exercise effects on corticospinal plasticity in relapsing remitting multiple sclerosis.

Expected benefit for participants
The benefits that you will have with your participation in this research study is the systematic evaluation of your clinical condition for a year, as well as your participation for a long time in an exercise program that will keep you physically active with possible improvement of your motor and cognitive functions and improving your quality of life.

Expected benefit for researchers and/or sponsors
All results from the study, will contribute to the scientific knowledge but also to the practical application by health professionals, regarding the effect of this type of movements and exercises in order to improve the current clinical situation of people with MS. Moreover, they will offer the advantage for better quality of rehabilitation in possible future progression of the disease.

Details of termination or early postponement of the research program.
All data that will collected from your assessments will be stored in the laboratory of the rehabilitation clinic, at the School of Health Sciences, of the CUT, as well as in the office of the senior physiotherapist of the Physiotherapy Unit, of CING. All the data that will be received within the specific research program will be destroyed after 5 years.

Description of procedures of handling data and/or biological samples of participants who withdraw from the study prior to its completion.
The data of the participants who will withdraw from the study before its completion will be destroyed immediately and will not be used in any study process. Specifically, any data stored on the researchers' computers will be deleted when you leave the study by the PI and any printed material collected will be destroyed and recycled.

Surname:	....	Name:	....
Signature:	....	Date:	....

CONSENT FORMS<br>for in a research program<br>(The forms are comprised of 8 pages)
**Title of the Programme you are invited to participate**
Investigation of in-phase bilateral exercise effects on corticospinal plasticity in relapsing remitting multiple sclerosis.

Full contact details and title of the person to whom participants can submit complaints or grievances regarding the program they participate in.
Dr. Charalambos Charalambous<br>Head of the department of research and international collaborations<br>Cyprus University of Technology<br>Telephone number: 00357 25 002562<br>email: c.chrisostomou@cut.ac.cy

Full contact details and title of the person whom participants can contact for more information or clarifications about the research program.
Dr. Nikos Konstantinou<br>Assistant Professor, Department of Rehabilitation Sciences, Cyprus University of Technology.<br>Vragadinou 15, Limassol, 3041<br>telephone number: 00357 25002294<br>email: nikos.konstantinou@cut.ac.cy

Surname:	....	Name:	....
Signature:	....	Date:	....

Appendix 2b_ Information sheet about TMS

- TMS is a non-invasive method of diagnosis and /or treatment of the motor cortex of the brain.

- The clinical use of TMS for diagnostic purposes is the ability to measure brain activity and connect specific areas of the nervous system.

- Throughout TMS it is possible to detect basic pathophysiological changes that cause disorders due to dysfunction of the motor cortex of the brain, resulting from certain diseases or injuries.

- In summary, the use of TMS for diagnostic purpose, is the investigation of the connection between the primary motor cortex (which is responsible for muscle movements) and the muscles, in order to assess the damage caused by stroke, diagnosis of multiple sclerosis and amyotrophic lateral sclerosis associated with neuronal death disorders, motor disorders, and general brain injuries.

- TMS affects the electrical activity of the brain through a pulsed magnetic field. The magnetic field is generated by transmitting current pulses through an insulated coil which has a circular or butterfly shape.

- The coil is wrapped in plastic and placed close to the scalp so that the magnetic field can focus on specific areas of the cerebral cortex.

- The magnetic field created in TMS, can penetrate the scalp without pain and safely to activate specific neurons (brain cells).

- The use of TMS has been extensively validated in clinical trials and medical research studies, and has been approved for use in various countries (eg, Canada since 2002 and later in the USA).

- Several scientific articles refer to the encouraging results of TMS in the treatment of Parkinson's disease, Multiple Sclerosis, Schizophrenia with Acoustic hallucinations, Rehabilitation after stroke and other emotional disorders.

Appendix 3_ Figure 1

BASELINE INTERVENTION FOLLOW-UP

**Participant A**	c n f	c n	c n			c	n	c	n	c	n	c	n	c	n f				c n f				c n f				c n f				
**Participant B**	c n f	c n	c n	c n			c	n	c	n	c	n	c	n	c	n f				c n f				c n f				c n f			
**Participant C**	c n f	c n	c n	c n	c n			c	n	c	n	c	n	c	n	c	n f				c n f				c n f				c n f		
**Participant D**	c n f	c n	c n	c n	c n	c n			c	n	c	n	c	n	c	n	c	n f				c n f				c n f				c n f	
**Participant E**	c n f	c n	c n	c n	c n	c n	c n			c	n	c	n	c	n	c	n	c	n f				c n f				c n f				c n f
**Weeks**	**1**	**2**	**3**	**4**	**5**	**6**	**7**	**8**	**9**	**10**	**11**	**12**	**13**	**14**	**15**	**16**	**17**	**18**	**19**	**20**	**21**	**22**	**23**	**24**	**25**	**26**	**27**	**28**	**29**	**30**	**31**

Timeline and schematic representation of the study's design. Grey colour represents the intervention phase. Each row (A-E) represents a different participant. (c) clinical assessment. (n) neurophysiological assessment via TMS. (f) Modified Fatigue Impact Scale questionnaire. Every cell represents a different week, so every procedure which is included (i.e., c, n, f) will be performed during the corresponding week but in different days.

References

1. Whitall J, McCombe Waller S, Sorkin JD, Forrester LW, Macko RF, Hanley DF, et al. Bilateral and unilateral arm training improve motor function through differing neuroplastic mechanisms: A single-blinded randomized controlled trial. Neurorehabil Neural Repair. 2011;25(2):118–29.

2. Smith AL, Richard Staines W. Cortical and behavioral adaptations in response to short-term inphase versus antiphase bimanual movement training. Exp Brain Res. 2010;205(4):465–77.

3. Neva JL, Legon W, Staines WR. Primary motor cortex excitability is modulated with bimanual training. Neurosci Lett [Internet]. 2012;514(2):147–51. Available from: http://dx.doi.org/10.1016/j.neulet.2012.02.075

4. Stinear JW, Byblow WD. Disinhibition in the human motor cortex is enhanced by synchronous upper limb movements. J Physiol. 2002;543(1):307–16.

5. Sandroff BM, Jones CD, Baird JF, Motl RW. Systematic Review on Exercise Training as a Neuroplasticity-Inducing Behavior in Multiple Sclerosis. Neurorehabil Neural Repair. 2020;34(7):575–88.

6. Reina-Gutiérrez S, Cavero-Redondo I, Martínez-Vizcaíno V, Núñez de Arenas-Arroyo S, López-Muñoz P, Álvarez-Bueno C, et al. The type of exercise most beneficial for quality of life in people with multiple sclerosis: A network meta-analysis. Ann Phys Rehabil Med. 2022;65(3).

7. Akbar N, Sandroff BM, Wylie GR, Strober LB, Smith A, Goverover Y, et al. Progressive resistance exercise training and changes in resting-state functional connectivity of the caudate in persons with multiple sclerosis and severe fatigue: A proof-of-concept study. Neuropsychol Rehabil [Internet]. 2020;30(1):54–66. Available from: https://doi.org/10.1080/09602011.2018.1449758

8. Proschinger S, Kuhwand P, Rademacher A, Walzik D, Warnke C, Zimmer P, et al. Fitness , physical activity , and exercise in multiple sclerosis : a systematic review on current evidence for interactions with disease activity and progression. J Neurol [Internet]. 2022;(January). Available from: https://doi.org/10.1007/s00415-021-10935-6

9. Kratochwill, T. R. Hitchcock, J. Horner, R. H. Levin, J. R. Odom, S. L. Rindskopf, D. M Shadish WR. Single-Case Design Technical Documentation. Work Clear website http//ies.ed.gov/ncee/wwc/pdf/wwc_scd.pdf. 2010;(December):2010.

10. Abbaspoor E, Zolfaghari M, Ahmadi B, Khodaei K. The effect of combined functional training on BDNF, IGF-1, and their association with health-related fitness in the multiple sclerosis women. Growth Horm IGF Res [Internet]. 2020;52(March):101320. Available from: https://doi.org/10.1016/j.ghir.2020.101320

11. Pascual-Leone a, Tarazona F, Keenan J, Tormos JM, Hamilton R, Catala MD.

Transcranial magnetic stimulation and neuroplasticity. Neuropsychologia [Internet]. 1999;37(2):207–17. Available from: http://www.ncbi.nlm.nih.gov/pubmed/10080378

12. Franchignoni F, Horak F, Godi M, Nardone A, Giordano A. Using psychometric techniques to improve the balance evaluation systems test: The mini-bestest. J Rehabil Med. 2010;42(4):323–31.

13. Nieuwenhuis MM, Tongeren H Van, Sørensen PS, Ravnborg M. The Six Spot Step Test : a new measurement for walking ability in multiple sclerosis. 2006;(September 2005).

14. Andrews AW, Thomas MW, Bohannon RW. Normative values for isometric muscle force measurements obtained with hand-held dynamometers. Phys Ther. 1996;76(3):248–59.

15. Benedict RHB, Deluca J, Phillips G, LaRocca N, Hudson LD, Rudick R. Validity of the Symbol Digit Modalities Test as a cognition performance outcome measure for multiple sclerosis. Mult Scler. 2017;23(5):721–33.

16. Fisk JD, Ritvo PG, Ross L, Haase DA, Marrie TJ, Schlech WF. Measuring the functional impact of fatigue: Initial validation of the fatigue impact scale. Clin Infect Dis. 1994;18:S79–83.

17. Zhan S, Ottenbacher KJ. Single subject research designs for disability research. Disabil Rehabil. 2001;23(1):1–8.

18. Lobo MA, Moeyaert M, Cunha AB, Babik I. Single-case design, analysis, and quality assessment for intervention research. J Neurol Phys Ther. 2017;41(3):187–97.

19. Lublin FD, Reingold SC. Defining the clinical course of multiple sclerosis: Results of an international survey. Neurology. 1996;46(4):907–11.

20. Moghaddam VK, Dickerson AS, Bazrafshan E, Seyedhasani SN. Socioeconomic determinants of global distribution of multiple sclerosis : an ecological investigation based on Global Burden of Disease data. 2021;1–11.

21. Walton C, King R, Rechtman L, Kaye W, Leray E, Marrie RA, et al. Rising prevalence of multiple sclerosis worldwide : Insights from the Atlas of MS , third edition. :1–6.

22. Dobson R, Giovannoni G. Multiple sclerosis – a review. Eur J Neurol. 2019;26(1):27–40.

23. Lunde HMB, Assmus J, Myhr KM, Bø L, Grytten N. Survival and cause of death in multiple sclerosis: A 60-year longitudinal population study. J Neurol Neurosurg Psychiatry. 2017;88(8):621–5.

24. Scalfari A, Knappertz V, Cutter G, Goodin DS, Ashton R, Ebers GC. Mortality in patients with multiple sclerosis. Neurology. 2013;81(2):184–92.

25. Kingwell E, Zhu F, Evans C, Duggan T, Oger J, Tremlett H. Causes that Contribute to the Excess Mortality Risk in Multiple Sclerosis: A Population-Based Study. Neuroepidemiology. 2020;54(2):131–9.

26. Maguire R, Maguire P. Caregiver Burden in Multiple Sclerosis: Recent Trends and Future Directions. Curr Neurol Neurosci Rep. 2020;20(7).

27. Kouzoupis AB, Paparrigopoulos T, Soldatos M, Papadimitriou GN. The family of the multiple sclerosis patient: A psychosocial perspective. Int Rev Psychiatry. 2010;22(1):83–9.

28. Lublin FD, Coetzee T, Cohen JA, Marrie RA, Thompson AJ. The 2013 clinical course descriptors for multiple sclerosis: A clarification. Neurology. 2020;94(24):1088–92.

29. Kister I, Bacon TE, Chamot E, Salter AR, Cutter GR, Kalina JT, et al. Multiple Sclerosis Symptoms. 2013;(June 2011):146–57.

30. Norbye AD, Midgard R, Thrane G. Spasticity, gait, and balance in patients with multiple sclerosis: A cross-sectional study. Physiother Res Int. 2020;25(1):1–9.

31. Benedict RHB, Amato MP, Deluca J, Geurts JJG. Cognitive impairment in multiple sclerosis : clinical management , MRI , and therapeutic avenues. Lancet Neurol [Internet]. 2020;19(10):860–71. Available from: http://dx.doi.org/10.1016/S1474-4422(20)30277-5

32. Frndak SE, Kordovski VM, Cookfair D, Rodgers JD, Weinstock-Guttman B, Benedict RHB. Disclosure of disease status among employed multiple sclerosis patients: Association with negative work events and accommodations. Mult Scler J. 2015;21(2):225–34.

33. Strober L, Chiaravalloti N, Moore N, Deluca J. Unemployment in multiple sclerosis (MS): Utility of the MS Functional Composite and cognitive testing. Mult Scler. 2014;20(1):112–5.

34. Kerbrat A, Gros C, Badji A, Bannier E, Galassi F, Labauge P, et al. Multiple sclerosis lesions in motor tracts from brain to cervical cord : spatial distribution and correlation with disability. 2020;2089–105.

35. Zackowski KM, Chodkowski BA, Calabresi PA. Corticospinal Tract Abnormalities Are Associated with Weakness in Multiple Sclerosis. 2008;333–9.

36. Tovar-moll F, Evangelou IE, Chiu AW, Auh S, Chen C, Ehrmantraut M, et al. Diffuse and Focal Corticospinal Tract Disease and Its Impact on Patient Disability in Multiple Sclerosis. 2014;14–6.

37. Fritz NE, Keller J, Calabresi PA, Zackowski KM. NeuroImage : Clinical Quantitative measures of walking and strength provide insight into brain corticospinal tract pathology in multiple sclerosis. NeuroImage Clin [Internet]. 2017;14:490–8. Available from: http://dx.doi.org/10.1016/j.nicl.2017.02.006

38. Pawlitzki M, Neumann J, Heidel J, Stadler E, Sweeney-reed C, Sailer M. Loss of corticospinal tract integrity in early MS disease stages. 2017;0.

39. Shanahan CJ, Walt A Van Der, Boonstra FMC, Glarin R, Kilpatrick TJ, Geurts JJG, et al.

B RAIN AIN COMMUNICATIONS Axonal loss in major sensorimotor tracts is associated with impaired motor performance in minimally disabled multiple sclerosis patients. 2021;

40. Lemon RN. Descending pathways in motor control. Annu Rev Neurosci. 2008;31(Cm):195–218.

41. Lipp I, Tomassini V. Neuroplasticity and motor rehabilitation in multiple sclerosis. 2015;6(March):1–3.

42. Flachenecker P. Clinical implications of neuroplasticity - the role of rehabilitation in multiple sclerosis. Front Neurol. 2015;6(MAR):1–4.

43. Tomassini V, Matthews PM, Thompson AJ, Fuglø D, Geurts JJ, Johansen-berg H, et al. Neuroplasticity and functional recovery in multiple sclerosis. Nat Publ Gr [Internet]. 2012;8(11):635–46. Available from: http://dx.doi.org/10.1038/nrneurol.2012.179

44. Tavazzi E, Cazzoli M, Pirastru A, Blasi V, Rovaris M, Bergsland N, et al. Neuroplasticity and Motor Rehabilitation in Multiple Sclerosis : A Systematic Review on MRI Markers of Functional and Structural Changes. 2021;15(October).

45. Mori F, Kusayanagi H, Nicoletti CG, Weiss S, Marciani MG, Centonze D. Cortical plasticity predicts recovery from relapse in multiple sclerosis. Mult Scler J. 2014;20(4):451–7.

46. Mori F, Rossi S, Piccinin S, Motta C, Mango D, Kusayanagi H, et al. Synaptic plasticity and PDGF signaling defects underlie clinical progression in multiple sclerosis. J Neurosci. 2013;33(49):19112–9.

47. Neva JL, Lakhani B, Brown KE, Wadden KP, Mang CS, Ledwell NHM, et al. Multiple measures of corticospinal excitability are associated with clinical features of multiple sclerosis. Behav Brain Res [Internet]. 2016;297:187–95. Available from: http://dx.doi.org/10.1016/j.bbr.2015.10.015

48. Zeller D, Classen J. Plasticity of the motor system in multiple sclerosis. Neuroscience [Internet]. 2014;283(June):222–30. Available from: http://dx.doi.org/10.1016/j.neuroscience.2014.05.043

49. Zentgraf K, Helm F. Brain Changes in Response to Exercise - Methodologies for Identifying the Physiological Effects of Physical Exercise. 2020;II:815–31.

50. Moucha R, Ã MPK. Cortical plasticity and rehabilitation. 2006;

51. Prosperini L, Filippo M Di. Beyond clinical changes: Rehabilitation-induced neuroplasticity in MS. Mult Scler J. 2019;25(10):1348–62.

52. Marta Niwald EM. Novel Physiotherapy Approach for Multiple Sclerosis. J Nov Physiother. 2014;04(05).

53. Diechmann MD, Campbell E, Coulter E, Paul L, Dalgas U, Hvid LG. Effects of exercise training on neurotrophic factors and subsequent neuroprotection in persons with multiple

sclerosis—a systematic review and meta-analysis. Brain Sci. 2021;11(11).

54. Learmonth YC, Motl RW. Exercise Training for Multiple Sclerosis : A Narrative Review of History , Benefits , Safety , Guidelines , and Promotion. 2021;

55. Sun Y, Zehr EP. Training-induced neural plasticity and strength are amplified after stroke. Exerc Sport Sci Rev. 2019;47(4):223–9.

56. Garry MI, van Steenis RE, Summers JJ. Interlimb coordination following stroke. Hum Mov Sci. 2005;24(5–6):849–64.

57. McCombe Waller S, Whitehall J. Bilateral arm training: Why and who benefits? NeuroRehabilitation. 2008;23:29–41.

58. Liepert J, Mingers D, Heesen C, Bäumer T, Weiller C. Motor cortex excitability and fatigue in multiple sclerosis: A transcranial magnetic stimulation study. Mult Scler. 2005;11(3):316–21.

59. Toyokura M, Muro I, Komiya T, Obara M. Activation of pre-supplementary motor area (SMA) and SMA proper during unimanual and bimanual complex sequences: An analysis using functional magnetic resonance imaging. J Neuroimaging. 2002;12(2):172–8.

60. Staines WR, McIlroy WE, Graham SJ, Black SE. Bilateral movement enhances ipsilesional cortical activity in acute stroke: A pilot functional MRI study [4] (multiple letters). Neurology. 2001;57(9):1740–1.

61. Calabrese M, Filippi M, Gallo P. Cortical lesions in multiple sclerosis. Nat Rev Neurol [Internet]. 2010;6(8):438–44. Available from: http://dx.doi.org/10.1038/nrneurol.2010.93

62. Tate RL, Perdices M, Rosenkoetter U, Wakim D, Godbee K, Togher L, et al. Revision of a method quality rating scale for single-case experimental designs and n-of-1 trials: The 15-item Risk of Bias in N-of-1 Trials (RoBiNT) Scale. Neuropsychol Rehabil. 2013;23(5):619–38.

63. JF K. Rating neurologic impairment in multiple sclerosis: an expanded disability status scale (EDSS). Neurology. 1983;Nov;33(11):1444-52.

64. Meseguer-Henarejos AB, SăNCHEZ-MECA J, López-Pina JA, CARLES-HERNăNDEZ R. Inter-and intra-rater reliability of the Modified Ashworth Scale: A systematic review and meta-analysis. Eur J Phys Rehabil Med. 2018;54(4):576–90.

65. Sangarapillai K, Norman BM, Almeida QJ. Boxing vs Sensory Exercise for Parkinson’s Disease: A Double-Blinded Randomized Controlled Trial. Neurorehabil Neural Repair. 2021;

66. DeLuca J, Chiaravalloti ND, Sandroff BM. Treatment and management of cognitive dysfunction in patients with multiple sclerosis. Nat Rev Neurol [Internet]. 2020;16(6):319–32. Available from: http://dx.doi.org/10.1038/s41582-020-0355-1

67. Kalb R, Brown TR, Coote S, Costello K, Dalgas U, Garmon E, et al. Exercise and lifestyle

physical activity recommendations for people with multiple sclerosis throughout the disease course. Mult Scler J. 2020;26(12):1459–69.

68. Groppa S, Oliviero A, Eisen A, Quartarone A, Cohen LG, Mall V, et al. A practical guide to diagnostic transcranial magnetic stimulation: Report of an IFCN committee. Clin Neurophysiol [Internet]. 2012;123(5):858–82. Available from: http://dx.doi.org/10.1016/j.clinph.2012.01.010

69. Rossini PM, Burke D, Chen R, Cohen LG, Daskalakis Z, Di Iorio R, et al. Non-invasive electrical and magnetic stimulation of the brain, spinal cord, roots and peripheral nerves: Basic principles and procedures for routine clinical and research application: An updated report from an I.F.C.N. Committee. Clin Neurophysiol [Internet]. 2015;126(6):1071–107. Available from: http://dx.doi.org/10.1016/j.clinph.2015.02.001

70. Pascual-Leone A, Dang N, Cohen LG, Brasil-Neto JP, Cammarota A, Hallett M. Modulation of muscle responses evoked by transcranial magnetic stimulation during the acquisition of new fine motor skills. J Neurophysiol. 1995;74(3):1037–45.

71. Charalambous CC, Dean JC, Adkins DAL, Hanlon CA, Bowden MG. Characterizing the corticomotor connectivity of the bilateral ankle muscles during rest and isometric contraction in healthy adults. J Electromyogr Kinesiol [Internet]. 2018;41(February):9–18. Available from: https://doi.org/10.1016/j.jelekin.2018.04.009

72. Rossini PM, Barker AT, Berardelli A, Caramia MD, Caruso G, Cracco RQ, et al. Non-invasive electrical and magnetic stimulation of the brain, spinal cord, roots and peripheral nerves: Basic principles and procedures for routine clinical and research application: An updated report from an I.F.C.N. Committee. Clin Neurophysiol. 1994;91(2):79–92.

73. Awiszus F. TMS and threshold hunting [Internet]. Vol. 56. Elsevier B.V.; 2003. 13–23 p. Available from: http://dx.doi.org/10.1016/S1567-424X(09)70205-3

74. Silbert BI, Patterson HI, Pevcic DD, Windnagel KA, Thickbroom GW. Clinical Neurophysiology A comparison of relative-frequency and threshold-hunting methods to determine stimulus intensity in transcranial magnetic stimulation. Clin Neurophysiol [Internet]. 2013;124(4):708–12. Available from: http://dx.doi.org/10.1016/j.clinph.2012.09.018

75. Goldsworthy MR, Hordacre B, Ridding MC. Minimum number of trials required for within- and between-session reliability of TMS measures of corticospinal excitability. Neuroscience [Internet]. 2016;320:205–9. Available from: http://dx.doi.org/10.1016/j.neuroscience.2016.02.012

76. Snow NJ, Wadden KP, Chaves AR, Ploughman M. Review Article Transcranial Magnetic Stimulation as a Potential Biomarker in Multiple Sclerosis : A Systematic Review with Recommendations for Future Research. 2019;2019.

77. Horak FB, Wrisley DM, Frank J. The balance evaluation systems test (BESTest) to differentiate balance deficits. Phys Ther. 2009;89(5):484–98.

78. Carpinella I, Cattaneo D, Ferrarin M. Quantitative assessment of upper limb motor function in Multiple Sclerosis using an instrumented Action Research Arm Test. J Neuroeng Rehabil. 2014;11(1):1–16.

79. Ziemann U, Reis J, Schwenkreis P, Rosanova M, Strafella A, Badawy R, et al. TMS and drugs revisited 2014. Clin Neurophysiol [Internet]. 2015;126(10):1847–68. Available from: http://dx.doi.org/10.1016/j.clinph.2014.08.028

80. Hess CW, Mills KR, Murray NM. Responses in small hand muscles from magnetic stimulation of the human brain. J Physiol. 1987;388(1):397–419.

81. Vanteemar S. Sreeraj S, Uvais2 NA, Mohanty3 S, Kumar3 S, Department. Indian nursing students' attitudes toward mental illness and persons with mental illness. Ind Psychiatry J. 2019;195–201.

82. Paulus W, Peterchev A V., Ridding M. Transcranial electric and magnetic stimulation: Technique and paradigms. Handb Clin Neurol. 2013;116(0):329–42.

83. Balloff C, Penner I-K, Ma M, Georgiades I, Scala L, Troullinakis N, et al. The degree of cortical plasticity correlates with cognitive performance in patients with Multiple Sclerosis. Brain Stimul [Internet]. 2022;15(2):403–13. Available from: https://doi.org/10.1016/j.brs.2022.02.007

84. Stampanoni Bassi M, Buttari F, Maffei P, De Paolis N, Sancesario A, Gilio L, et al. Practice-dependent motor cortex plasticity is reduced in non-disabled multiple sclerosis patients. Clin Neurophysiol [Internet]. 2020;131(2):566–73. Available from: https://doi.org/10.1016/j.clinph.2019.10.023

85. Hallett M. Transcranial Magnetic Stimulation: A Primer. Neuron. 2007;55(2):187–99.

86. Zimnowodzki S, Butrum M, Kimura J, Stålberg E, Mahajan S, Gao L. Emergence of F-waves after repetitive nerve stimulation. Clin Neurophysiol Pract [Internet]. 2020;5:100–3. Available from: https://doi.org/10.1016/j.cnp.2020.04.002

87. Heyvaert M, Onghena P. Analysis of single-case data: Randomisation tests for measures of effect size. Neuropsychol Rehabil. 2014;24(3–4):507–27.

88. Krasny-Pacini A, Evans J. Single-case experimental designs to assess intervention effectiveness in rehabilitation: A practical guide. Ann Phys Rehabil Med [Internet]. 2018;61(3):164–79. Available from: http://dx.doi.org/10.1016/j.rehab.2017.12.002

1. Whitall, J.; McCombe Waller, S.; Sorkin, J.D.; Forrester, L.W.; Macko, R.F.; Hanley, D.F.; Goldberg, A.P.; Luft, A. Bilateral and Unilateral Arm Training Improve Motor Function through Differing Neuroplastic Mechanisms: A Single-Blinded Randomized Controlled Trial. *Neurorehabil. Neural Repair* **2011**, *25*, 118–129, doi:10.1177/1545968310380685.

2. Smith, A.L.; Richard Staines, W. Cortical and Behavioral Adaptations in Response to Short-Term Inphase versus Antiphase Bimanual Movement Training. *Exp. Brain Res.* **2010**, *205*, 465–477, doi:10.1007/s00221-010-2381-5.

3. Neva, J.L.; Legon, W.; Staines, W.R. Primary Motor Cortex Excitability Is Modulated with Bimanual Training. *Neurosci. Lett.* **2012**, *514*, 147–151, doi:10.1016/j.neulet.2012.02.075.

4. Stinear, J.W.; Byblow, W.D. Disinhibition in the Human Motor Cortex Is Enhanced by Synchronous Upper Limb Movements. *J. Physiol.* **2002**, *543*, 307–316, doi:10.1113/jphysiol.2002.023986.

5. Sandroff, B.M.; Jones, C.D.; Baird, J.F.; Motl, R.W. Systematic Review on Exercise Training as a Neuroplasticity-Inducing Behavior in Multiple Sclerosis. *Neurorehabil. Neural Repair* **2020**, *34*, 575–588, doi:10.1177/1545968320921836.

6. Reina-Gutiérrez, S.; Cavero-Redondo, I.; Martínez-Vizcaíno, V.; Núñez de Arenas-Arroyo, S.; López-Muñoz, P.; Álvarez-Bueno, C.; Guzmán-Pavón, M.J.; Torres-Costoso, A. The Type of Exercise Most Beneficial for Quality of Life in People with Multiple Sclerosis: A Network Meta-Analysis. *Ann. Phys. Rehabil. Med.* **2022**, *65*, doi:10.1016/j.rehab.2021.101578.

7. Akbar, N.; Sandroff, B.M.; Wylie, G.R.; Strober, L.B.; Smith, A.; Goverover, Y.; Motl, R.W.; DeLuca, J.; Genova, H. Progressive Resistance Exercise Training and Changes in Resting-State Functional Connectivity of the Caudate in Persons with Multiple Sclerosis and Severe Fatigue: A Proof-of-Concept Study. *Neuropsychol. Rehabil.* **2020**, *30*, 54–66, doi:10.1080/09602011.2018.1449758.

8. Proschinger, S.; Kuhwand, P.; Rademacher, A.; Walzik, D.; Warnke, C.; Zimmer, P.; Joisten, N. Fitness , Physical Activity , and Exercise in Multiple Sclerosis : A Systematic Review on Current Evidence for Interactions with Disease Activity and Progression. *J. Neurol.* **2022**, doi:10.1007/s00415-021-10935-6.

9. Kratochwill, T. R. Hitchcock, J. Horner, R. H. Levin, J. R. Odom, S. L. Rindskopf, D. M Shadish, W.R. Single-Case Design Technical Documentation. *What Work. Clear.* **2010**, 2010.

10. Abbaspoor, E.; Zolfaghari, M.; Ahmadi, B.; Khodaei, K. The Effect of Combined Functional Training on BDNF, IGF-1, and Their Association with Health-Related Fitness in the Multiple Sclerosis Women. *Growth Horm. IGF Res.* **2020**, *52*, 101320, doi:10.1016/j.ghir.2020.101320.

11. Pascual-Leone, a; Tarazona, F.; Keenan, J.; Tormos, J.M.; Hamilton, R.; Catala, M.D. Transcranial Magnetic Stimulation and Neuroplasticity. *Neuropsychologia* **1999**, *37*, 207–

217, doi:S0028393298000955 [pii].

12. Franchignoni, F.; Horak, F.; Godi, M.; Nardone, A.; Giordano, A. Using Psychometric Techniques to Improve the Balance Evaluation Systems Test: The Mini-Bestest. *J. Rehabil. Med.* **2010**, *42*, 323–331, doi:10.2340/16501977-0537.

13. Nieuwenhuis, M.M.; Tongeren, H. Van; Sørensen, P.S.; Ravnborg, M. The Six Spot Step Test : A New Measurement for Walking Ability in Multiple Sclerosis. *Mult. Scler. J.* **2006**.

14. Andrews, A.W.; Thomas, M.W.; Bohannon, R.W. Normative Values for Isometric Muscle Force Measurements Obtained with Hand-Held Dynamometers. *Phys. Ther.* **1996**, *76*, 248–259, doi:10.1093/ptj/76.3.248.

15. Benedict, R.H.B.; Deluca, J.; Phillips, G.; LaRocca, N.; Hudson, L.D.; Rudick, R. Validity of the Symbol Digit Modalities Test as a Cognition Performance Outcome Measure for Multiple Sclerosis. *Mult. Scler.* **2017**, *23*, 721–733, doi:10.1177/1352458517690821.

16. Fisk, J.D.; Ritvo, P.G.; Ross, L.; Haase, D.A.; Marrie, T.J.; Schlech, W.F. Measuring the Functional Impact of Fatigue: Initial Validation of the Fatigue Impact Scale. *Clin. Infect. Dis.* **1994**, *18*, S79–S83, doi:10.1093/clinids/18.Supplement_1.S79.

17. Zhan, S.; Ottenbacher, K.J. Single Subject Research Designs for Disability Research. *Disabil. Rehabil.* **2001**, *23*, 1–8, doi:10.1080/09638280150211202.

18. Pascual-Leone, A.; Tarazona, F.; Keenan, J.; Tormos, J.M.; Hamilton, R.; Catala, M.D. Transcranial Magnetic Stimulation and Neuroplasticity. *Neuropsychologia* **1998**, *37*, 207–217, doi:10.1016/S0028-3932(98)00095-5.

19. Lobo, M.A.; Moeyaert, M.; Cunha, A.B.; Babik, I. Single-Case Design, Analysis, and Quality Assessment for Intervention Research. *J. Neurol. Phys. Ther.* **2017**, *41*, 187–197, doi:10.1097/NPT.0000000000000187.

1. Whitall, J.; McCombe Waller, S.; Sorkin, J.D.; Forrester, L.W.; Macko, R.F.; Hanley, D.F.; Goldberg, A.P.; Luft, A. Bilateral and Unilateral Arm Training Improve Motor Function through Differing Neuroplastic Mechanisms: A Single-Blinded Randomized Controlled Trial. *Neurorehabil. Neural Repair* **2011**, *25*, 118–129, doi:10.1177/1545968310380685.

2. Smith, A.L.; Richard Staines, W. Cortical and Behavioral Adaptations in Response to Short-Term Inphase versus Antiphase Bimanual Movement Training. *Exp. Brain Res.* **2010**, *205*, 465–477, doi:10.1007/s00221-010-2381-5.

3. Neva, J.L.; Legon, W.; Staines, W.R. Primary Motor Cortex Excitability Is Modulated

with Bimanual Training. *Neurosci. Lett.* **2012**, *514*, 147–151, doi:10.1016/j.neulet.2012.02.075.

4. Stinear, J.W.; Byblow, W.D. Disinhibition in the Human Motor Cortex Is Enhanced by Synchronous Upper Limb Movements. *J. Physiol.* **2002**, *543*, 307–316, doi:10.1113/jphysiol.2002.023986.

5. Sandroff, B.M.; Jones, C.D.; Baird, J.F.; Motl, R.W. Systematic Review on Exercise Training as a Neuroplasticity-Inducing Behavior in Multiple Sclerosis. *Neurorehabil. Neural Repair* **2020**, *34*, 575–588, doi:10.1177/1545968320921836.

6. Reina-Gutiérrez, S.; Cavero-Redondo, I.; Martínez-Vizcaíno, V.; Núñez de Arenas-Arroyo, S.; López-Muñoz, P.; Álvarez-Bueno, C.; Guzmán-Pavón, M.J.; Torres-Costoso, A. The Type of Exercise Most Beneficial for Quality of Life in People with Multiple Sclerosis: A Network Meta-Analysis. *Ann. Phys. Rehabil. Med.* **2022**, *65*, doi:10.1016/j.rehab.2021.101578.

7. Akbar, N.; Sandroff, B.M.; Wylie, G.R.; Strober, L.B.; Smith, A.; Goverover, Y.; Motl, R.W.; DeLuca, J.; Genova, H. Progressive Resistance Exercise Training and Changes in Resting-State Functional Connectivity of the Caudate in Persons with Multiple Sclerosis and Severe Fatigue: A Proof-of-Concept Study. *Neuropsychol. Rehabil.* **2020**, *30*, 54–66, doi:10.1080/09602011.2018.1449758.

8. Proschinger, S.; Kuhwand, P.; Rademacher, A.; Walzik, D.; Warnke, C.; Zimmer, P.; Joisten, N. Fitness , Physical Activity , and Exercise in Multiple Sclerosis : A Systematic Review on Current Evidence for Interactions with Disease Activity and Progression. *J. Neurol.* **2022**, doi:10.1007/s00415-021-10935-6.

9. Kratochwill, T. R. Hitchcock, J. Horner, R. H. Levin, J. R. Odom, S. L. Rindskopf, D. M Shadish, W.R. Single-Case Design Technical Documentation. *What Work. Clear.* **2010**, 2010.

10. Abbaspoor, E.; Zolfaghari, M.; Ahmadi, B.; Khodaei, K. The Effect of Combined Functional Training on BDNF, IGF-1, and Their Association with Health-Related Fitness in the Multiple Sclerosis Women. *Growth Horm. IGF Res.* **2020**, *52*, 101320, doi:10.1016/j.ghir.2020.101320.

11. Pascual-Leone, A.; Tarazona, F.; Keenan, J.; Tormos, J.M.; Hamilton, R.; Catala, M.D. Transcranial Magnetic Stimulation and Neuroplasticity. *Neuropsychologia* **1998**, *37*, 207–217, doi:10.1016/S0028-3932(98)00095-5.

12. Pascual-Leone, a; Tarazona, F.; Keenan, J.; Tormos, J.M.; Hamilton, R.; Catala, M.D. Transcranial Magnetic Stimulation and Neuroplasticity. *Neuropsychologia* **1999**, *37*, 207–217, doi:S0028393298000955 [pii].

13. Franchignoni, F.; Horak, F.; Godi, M.; Nardone, A.; Giordano, A. Using Psychometric Techniques to Improve the Balance Evaluation Systems Test: The Mini-Bestest. *J. Rehabil. Med.* **2010**, *42*, 323–331, doi:10.2340/16501977-0537.

14. Nieuwenhuis, M.M.; Tongeren, H. Van; Sørensen, P.S.; Ravnborg, M. The Six Spot Step Test : A New Measurement for Walking Ability in Multiple Sclerosis. *Mult. Scler. J.* **2006**.

15. Andrews, A.W.; Thomas, M.W.; Bohannon, R.W. Normative Values for Isometric Muscle Force Measurements Obtained with Hand-Held Dynamometers. *Phys. Ther.* **1996**, *76*, 248–259, doi:10.1093/ptj/76.3.248.

16. Benedict, R.H.B.; Deluca, J.; Phillips, G.; LaRocca, N.; Hudson, L.D.; Rudick, R. Validity of the Symbol Digit Modalities Test as a Cognition Performance Outcome Measure for Multiple Sclerosis. *Mult. Scler.* **2017**, *23*, 721–733, doi:10.1177/1352458517690821.

17. Fisk, J.D.; Ritvo, P.G.; Ross, L.; Haase, D.A.; Marrie, T.J.; Schlech, W.F. Measuring the Functional Impact of Fatigue: Initial Validation of the Fatigue Impact Scale. *Clin. Infect. Dis.* **1994**, *18*, S79–S83, doi:10.1093/clinids/18.Supplement_1.S79.

18. Zhan, S.; Ottenbacher, K.J. Single Subject Research Designs for Disability Research. *Disabil. Rehabil.* **2001**, *23*, 1–8, doi:10.1080/09638280150211202.

19. Lobo, M.A.; Moeyaert, M.; Cunha, A.B.; Babik, I. Single-Case Design, Analysis, and Quality Assessment for Intervention Research. *J. Neurol. Phys. Ther.* **2017**, *41*, 187–197, doi:10.1097/NPT.0000000000000187.
